# Polyphosphate modulates the stress-responsive formation of functional RNA-protein condensates in bacteria and mammalian cells

**DOI:** 10.1371/journal.pbio.3003775

**Published:** 2026-04-27

**Authors:** Jian Guan, Rebecca Lee Hurto, Akash Rai, Janakraj Bhattrai, Christopher A. Azaldegui, Luis A. Ortiz-Rodríguez, Quancheng Liu, Julie S. Biteen, Lydia Freddolino, Ursula Jakob

**Affiliations:** 1 Department of Molecular, Cellular and Developmental Biology, University of Michigan, Ann Arbor, Michigan, United States of America; 2 Department of Biological Chemistry, University of Michigan, Ann Arbor, Michigan, United States of America; 3 Program in Chemical Biology, University of Michigan, Ann Arbor, Michigan, United States of America; 4 Department of Chemistry, University of Michigan, Ann Arbor, Michigan, United States of America; 5 Department of Computational Medicine and Bioinformatics, University of Michigan, Ann Arbor, Michigan, United States of America; National Centre for Biological Sciences, INDIA

## Abstract

Uncovering what drives select biomolecules to form phase-separated condensates in vivo and identifying their physiological significance are topics of fundamental importance. Here, we show that nitrogen-starved *Escherichia coli* produces long-chain polyphosphates, which scaffold the RNA chaperone Hfq into high molecular weight complexes, which eventually phase separate together with components of the RNA translation and processing machinery. The presence of polyphosphate within these condensates controls Hfq function by selectively stabilizing polyadenylated RNAs involved in transcription and protein translation and by promoting interactions with translation- and RNA-metabolism-associated proteins involved in de novo protein synthesis. Lack of polyphosphate significantly impairs condensate formation, increases cell death, and hinders recovery from N-starvation. In functional analogy, we demonstrate that polyP contributes specifically to the formation of Processing (P)-bodies in mammalian cell lines, revealing that a single, highly conserved and ancestral polyanion serves as a modulator for functional phase-separated condensate formation across the tree of life.

## Abstract 2

The RNA chaperone Hfq is known to assemble into biomolecular condensates in response to stress in E.coli, but what leads to Hfq’s phase separation is not clear. This study shows that polyphosphate serves as a scaffold for the formation of condensates with Hfq, which then sequester and selectively stabilize polyadenylated transcripts critical for the recovery from nitrogen starvation.

## Introduction

Membrane-less organelles, also referred to as biomolecular condensates, arise from liquid–liquid phase separation (LLPS), where biomolecules in a homogeneous solution separate into a condensed liquid phase surrounded by a dilute phase [1,2]. Condensates serve as a critical addition to the endomembrane system in the subcellular organization of physiological processes as they promote specific segregation of biochemical pathways and local enrichment of cellular components [3]. The rapid formation of condensates and its independence from membrane synthesis provides cells with rapid and effective means to reorganize cellular processes in response to stress [4]. Indeed, several stress conditions lead to functional condensate formation in eukaryotes and, as shown more recently, also in prokaryotes [5]. For instance, oxidative stress, such as that elicited by arsenite treatment, triggers the formation of cytosolic stress granules (SGs) in mammalian cells, which sequester essential components of the protein translation machinery to globally prevent translation initiation during stress [6,7]. Similarly, select stress conditions increase the size and number of *P*rocessing (P)-bodies in mammalian cells or *B*acterial *R*ibonucleoprotein (BR)-bodies in *Caulobacter crescentus* [8–11], both types of condensates aiding in RNA surveillance and selective RNA degradation [8,12]. Yet, in none of these cases, the precise mechanism that leads to the rapid stress-induced formation and/or expansion of functional biomolecular condensates is fully understood.

Recent studies in *Escherichia coli* revealed that in response to nitrogen (N) starvation, hyperosmolarity or stationary growth, the conserved RNA chaperone Hfq assembles into a single, polar-localized condensate (i.e., H-body) [13–16]. Hfq is a small hexameric protein that shares structural features with eukaryotic LSm proteins, one of the major components of P-bodies [17]. Best known for its function as an RNA chaperone, Hfq facilitates sRNA-mRNA pairing, thus regulating mRNA stability and translation [18,19]. In addition, about 10%–20% of cellular Hfq is nucleoid-associated and involved in the silencing of mobile genetic elements [20–22]. Based on the observed recruitment of members of the RNA degradosome to the condensates, Hfq foci have been suggested to be critical for post-transcriptional gene regulation [13,16,23,24]. Yet, what leads to Hfq’s phase separation under stress conditions that stall de novo protein synthesis is not known. A recent study revealed that the sugar regulator TmaR, which itself forms a polar-localized condensate in *E. coli*, is necessary for H-body formation [16,23]. However, because TmaR condensates are also present under non-stress conditions [23,25], it is unlikely that this protein directly initiates Hfq foci formation during stress. Given the known involvement of polyanions in condensate formation [26–28], we thus wondered whether polyphosphate (polyP), the most ancient, ubiquitous, and highly negatively charged polymer known, might contribute to stress-induced Hfq foci formation [29]. We based this reasoning on the fact that stress conditions that cause Hfq condensate formation also trigger the accumulation of long-chain polyP [30] as well as on the recent finding that polyP supports the phase separation of Hfq in vitro and Hfq functionality in vivo [22]. Here, we demonstrate that the stress-induced formation of long-chain polyPs plays an important role in Hfq foci formation. The ability of polyP to scaffold Hfq into high molecular weight (HMW) assemblies appears to reduce the saturation concentration (*c*_*sat*_) of Hfq for phase separation, thus promoting liquid droplet formation at existing Hfq levels. We find that Hfq-polyP condensates sequester and selectively stabilize polyadenylated transcripts that are critical for bacterial survival of and recovery from N starvation. Extending to the extreme opposite end of the tree of life, we demonstrate that polyP accumulation also contributes to the formation of mammalian P-bodies but not SGs. Based on these findings, we propose that polyP plays a conserved role in shaping functional condensates throughout evolution, marking it as one of the most ancient modulators of condensate formation across biology.

## Results

### Loss of polyphosphate negatively affects stress-induced Hfq foci formation

**Fig 1:**
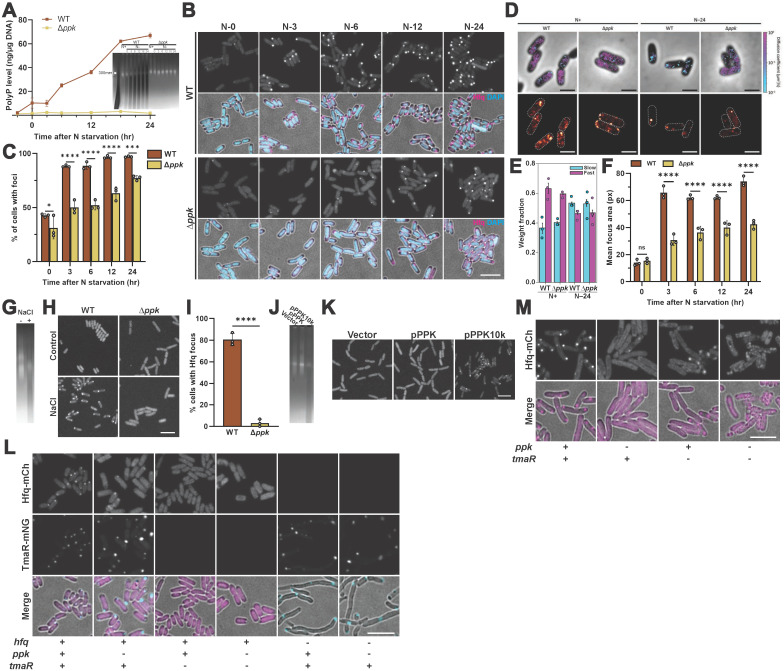
PolyP drives foci formation of Hfq in *Escherichia coli.* **(A)** Normalized polyP levels in WT (MG1655) and Δ*ppk E. coli* 2 h before and select timepoints upon entry into N-starvation (N-0). Error bars indicate SD (*n* = 3). **Inset:** PolyP extracted under the same conditions and visualized on TBE gel by DAPI staining and photobleaching, which will appear bright for nucleic acid and dark for polyP. Purified polyP 300mer was used as reference (lane 1). **(B)** Subcellular localization of Hfq-mCherry (magenta) and nucleoid (stained by DAPI, cyan) in *hfq::hfq-mCherry* WT (MG1655) or Δ*ppk* strains at select times (in hours) after the onset of N-starvation (N-). **(C)** Quantification of cells containing visible Hfq-mCherry foci after entering N-starvation. A minimum of 200 cells were quantified for each of three replicates under each time point. Comparisons were made by two-way ANOVA. Error bars show SD. *p < 0.05, ***p < 0.001, ****p < 0.0001. **(D)** Upper panel: Representative single molecule trajectories of Hfq-PAmCherry in indicated strains and conditions overlaid with phase contrast images. Trajectories are color-coded based on the apparent diffusion coefficient. Lower panel: Reconstructed super-resolution PALM images based on images above. Cell boundaries are indicated by dashed lines. Scale bars: 2 µm. **(E)** Percentage of single-molecule Hfq-PAmCherry trajectories in the fast (magenta) and slow (cyan) populations for cells in each of the indicated conditions. Data points are shown for each of at least three biological replicates. **(F)** Mean area of Hfq foci for cells under each of the indicated conditions; data points are shown for each of three biological replicates, with each such data point arising from joint quantitation of four imaging fields at each time point; see Methods for details. Comparisons were made by two-way ANOVA. Error bars show SD. ****p < 0.0001. **(G)** PolyP extracted from *E. coli* MG1655 *hfq::hfq-mCherry* after 4 h of mock treatment (−) or upon osmotic stress treatment with 1.17 M NaCl (+) in LB medium was visualized on TBE gel by DAPI staining and subsequent photobleaching. **(H)** Subcellular localization of Hfq-mCherry in *hfq::hfq-mCherry* WT (MG1655) or Δ*ppk* after 4 h of osmotic stress as in **(G)**. **(I)** Quantification of cells containing visible Hfq-mCherry foci after osmotic stress. At least 100 cells were quantified in each of three replicates. Comparisons were made by unpaired *t*-tests. Error bars indicate SD. ****p < 0.0001. **(J)** PolyP ex*t*racted from exponentially growing Δ*ppk E. coli hfq::hfq-mCherry* carrying an empty plasmid or a plasmid expressing WT *ppk* or *ppk10k* was visualized on TBE gel by DAPI staining and subsequent photobleaching, which darkens the polyP signal. **(K)** Subcellular localization of Hfq-mCherry in strains shown in (H). **(L)** Subcellular localization of Hfq-mCherry (magenta) and/or TmaR-mNG (cyan) in indicated *E. coli* strains at N-24. **(M)** Subcellular localization of Hfq-mCherry in indicated *E. coli* strains after 4 h of osmotic stress as in **(G)**. Scale bars: 5 µm unless otherwise indicated. All underlying data can be found in S1 Data.

To investigate the potential influence of polyP on Hfq foci formation in vivo, we replaced the chromosomal copy of *hfq* with a fully functional and previously validated *hfq-mCherry* gene fusion [16] in either *E. coli* MG1655 wild type (WT) or a mutant strain that lacks polyphosphate kinase (Ppk), the only known polyP-synthesizing enzyme in *E. coli* [31]. When grown in defined media supplemented with limiting amounts of nitrogen (N), both strains enter N-starvation at ~5 h (N-0) (S1A Fig). At this point, WT bacteria begin to accumulate polyP in the form of very long (>300 P_i_ units) chains (i.e., polyP-300) (Fig 1A). Within 3 h of N-starvation (N-3), over 80% and within 24 h (N-24), close to 100% of all WT bacteria contain one polarly localized Hfq focus (Fig 1B and 1C). Incubation of N-24 WT cells with 1,6-hexanediol, a known disrupter of in vivo protein condensates, dissolves these foci, consistent with their previously reported liquid–liquid phase properties (S1B Fig) [32]. In contrast, the Δ*ppk* strain, which does not produce any detectable polyP (Fig 1A), shows a significant delay in the appearance of Hfq foci with only ~50% of bacteria containing a detectable focus at N-6 (i.e., 6 h after onset of N-starvation) and ~75% of cells presenting foci at N-24 (Fig 1B and 1C). Single-molecule trajectory measurements of Hfq endogenously labeled with photoactivatable mCherry (PAmCherry) confirmed these results by revealing that, though the Hfq molecules have the same dynamics in N-24 Δ*ppk* cells compared to Hfq molecules in N-24 WT *E. coli*, these slow-moving molecules are confined to a single focus in WT cells but spread out throughout the cell in *Δppk* cells (Fig 1D and 1E). Moreover, incubation with 1,6-hexanediol caused a faster disappearance of Hfq foci in the *ppk* deletion cells as compared to WT cells, suggestive of fluidic and/or compositional differences between the Hfq foci formed in the presence and absence of polyP (S1B Fig). Automated image analysis also revealed that Hfq foci formed in the Δ*ppk* strain are, on average, between 40% and 50% smaller than foci formed in the WT strain (Fig 1F). These latter results were particularly surprising given that the N-24 Δ*ppk* strain accumulates almost twice the amount of total Hfq compared to N-24 WT *E. coli* (S1C Fig). Consistent with these results, the ratio of Hfq intensity within the foci *versus* the cellular background in the Δ*ppk* strain was reproducibly about 60% of the WT ratio at all measured times during N-starvation (S1D Fig). We also observed significant differences in focus formation between WT and Δ*ppk* strains when we expressed a 3× FLAG-tagged Hfq instead of Hfq-mCherry. Upon fixing and staining the bacteria with anti-FLAG antibodies, we detected focal accumulations of Hfq in about 30% of WT bacteria (consistent with published results [16]) as compared to less than 2% in Δ*ppk* bacteria (S1E and S1F Fig). Based on these results, we concluded that the Hfq foci that form in response to N-starvation in the absence of polyP are fewer, smaller, less intense and more 1,6-hexanediol sensitive than Hfq foci that form in the presence of polyP.

### Increased polyP levels are sufficient for Hfq foci formation

We next wondered whether the decrease in foci number and size that we observed in the N-24 *ppk* deletion mutant is directly caused by a lack of polyP or due to potentially increased levels of cellular ATP, the substrate of *E. coli* polyphosphate kinase and a known biological hydrotope that prevents condensate formation in vitro [33]. However, comparison of ATP concentrations in WT and *ppk* deletion bacteria revealed that the N-24 WT bacteria had even slightly higher levels of ATP compared to the N24 Δ*ppk* mutant, making this explanation unlikely (S1G Fig). A second stress condition that has been shown to cause a significant increase in Hfq foci formation is exposure to osmotic stress. We chose 4h of treatment with 1.17 M NaCl because these conditions had previously been shown to significantly increase polyP [30]. We confirmed those results and found a substantial accumulation of long and medium-chain polyP in these bacteria (Fig 1G). Of note, under these stress conditions, absence of *ppk* had even more dramatic effects; whereas ~80% of WT *E. coli* cells displayed a single, clearly visible and polar-localized Hfq focus, fewer than 5% of the Δ*ppk* bacteria showed any detectable focal accumulation of Hfq (Fig 1H and 1I). These results now raised the interesting possibility that Hfq foci formation might be directly linked to the stress-induced accumulation of polyP. To investigate whether this is indeed the case, we expressed a plasmid-encoded polyP-overproducing mutant variant Ppk10k [34] in the Δ*ppk* strain and compared Hfq foci formation with strains expressing either the vector alone or vector-encoded WT Ppk. We confirmed that the expression of Ppk10k causes a substantial increase in the steady state levels of polyP under exponential growth conditions compared to WT Ppk-expressing bacteria (Fig 1J). More relevant, however, we observed that most of the Ppk10k expressing cells contain one Hfq focus whereas neither of the other two Δ*ppk* strains shows any detectable Hfq foci under these growth conditions (Fig 1K). Based on these results, we concluded that the stress-induced increase in cellular polyP concentrations contributes to the phase separation of Hfq during N-starvation and osmotic stress.

### Both polyP and TmaR are necessary for Hfq foci formation during N-starvation

Previous studies reported that the formation of Hfq condensates in response to N-starvation, stationary growth or osmotic stress depends on the bacterial sugar metabolism regulator TmaR, which forms co-condensates with Hfq [16,25]. Intriguingly, however, these studies also revealed that before stress, significantly more bacteria harbor TmaR foci than Hfq foci, and that during N-starvation, the formation of Hfq foci lags significantly behind the increase in TmaR foci [16,23]. These results led to the conclusion that TmaR condensates, while necessary for Hfq foci formation, might not be sufficient and that other, potentially metabolic changes act downstream of TmaR to promote Hfq condensation [23]. We now wondered whether polyP is one such downstream factor, which would make the stress-induced synthesis of polyP likely a rate-limiting step for Hfq foci formation. Alternatively, it was also conceivable that polyP impacts condensation of TmaR, thereby only indirectly affecting Hfq phase separation. To distinguish between these possibilities, we expressed Hfq-mCherry in Δ*tmaR* and Δ*tmaR/*Δ*ppk* strains as well as TmaR-mNeonGreen (mNG) in WT, Δ*ppk*, Δ*hfq* and Δ*hfq/*Δ*ppk* strains and monitored stress-induced Hfq and/or TmaR foci formation. As expected, the formation of Hfq foci under N-starvation conditions was significantly impaired in the Δ*ppk* strain and lost in the Δ*tmaR* and Δ*tmaR/*Δ*ppk* strains (Fig 1L), the latter also not rescuable by the expression of Ppk10k (S1H Fig). In contrast, lack of polyP, Hfq or both did not appear to affect the overall number of TmaR foci but caused heterogeneity in the size and intensity of individual TmaR foci (Fig 1L). While these results suggested some level of feedback regulation between Hfq and/or polyP on TmaR condensate formation, they also implied that polyP acts more likely downstream of TmaR and hence directly on Hfq. These conclusions were further supported when we compared Hfq focus formation under strong osmotic stress conditions, where, as noted above (Fig 1G) deletion of *ppk* caused an over 90% reduction in Hfq focus formation, whereas deletion of *tmaR* only led to a ~60% reduction in visible Hfq foci (Figs 1M and S1I). The Δ*ppk/*Δ*tmaR* strain revealed a highly aberrant cellular Hfq distribution, distinctly different from the Hfq distribution in the individual *ppk* and *tmaR* deletion mutants. Our observations differ from the earlier study by Goldberger and colleagues [16], where the authors reported that deletion of *tmaR* resulted in complete loss of Hfq foci when bacteria were treated with 0.3 M NaCl. However, it should be noted that we used much higher NaCl concentrations (i.e., 1.17 M) to induce osmotic stress because these concentrations trigger substantial polyP accumulation while lower NaCl concentrations fail to do so (S1J Fig). We therefore conclude that under mild osmotic stress conditions (i.e., presence of 300 mM NaCl), TmaR is likely the main factor supporting Hfq foci formation. At higher osmotic stress conditions, however, TmaR and polyP appear to act as independent entities, both necessary yet individually not sufficient for the proper generation of in vivo condensates of Hfq.

### PolyP regulates the subcellular organization of Hfq during and after N-starvation

**Fig 2:**
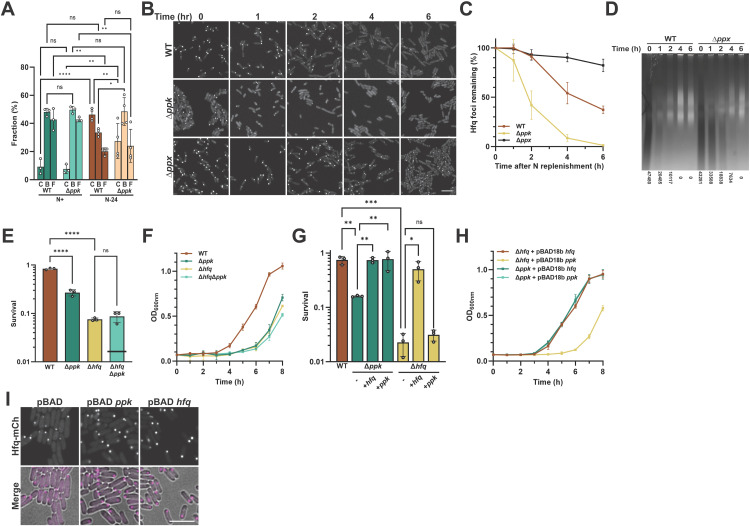
In vivo interplay between Hfq and polyP during N-starvation. **(A)** Fraction of Hfq-PAmCherry present in condensates (C), found in the nucleoid-bound (B) form or free (F) in the cytosol of N+ and N-24 WT (MG1655) or *ppk* deletion strains as assessed by single-molecule localization. >1,500 trajectories were analyzed per replicate (*N* ≥ 3) of each condition. Comparisons were made by 2-way ANOVA (*n* ≥ 3), *p < 0.05, **p < 0.01, ****p < 0.0001. Error bars indicate SD. **(B)** Subcellular localization of Hfq-mCherry in *hfq::hfq-mCherry* N-24 WT (MG1655), Δ*ppk,* or Δ*ppx Escherichia coli* at indicated time points after supplementing the media with fresh N source. **(C)** Percentage of Hfq-mCherry foci remaining calculated from **(B)**. The number of condensates at N-24 (i.e., *t* = 0 h) is set to 100% and used as a reference point. A minimum of 200 cells were quantified for each of three replicates under each time point, error bars indicate SD. **(D)** PolyP levels at the indicated timepoints after supplementing the growth media of N-24 *hfq::hfq-mCherry* WT (MG1655) or Δ*ppx* with fresh N source. PolyP was visualized on a TBE gel using DAPI staining and subsequent photobleaching, which darkens the polyP signal. Relative polyP levels (A.U.) are shown below. **(E)** Survival of WT (MG1655), Δ*ppk*, Δ*hfq*, and Δ*hfq*Δ*ppk E. coli* at N48. Similar results were obtained in the respective MG1655 *hfq::hfq-mCherry* strains. The black bar in the double mutant indicates the expected phenotype if *ppk* and *hfq* deletion behaved log-additively. **(F)** Recovery growth of N-24 WT, Δ*ppk*, Δ*hfq*, and Δ*hfq*Δ*ppk E. coli* after dilution into fresh Gutnick medium supplemented with 3 mM NH_4_Cl. **(G)** Survival of WT (MG1655), Δ*ppk*, or Δ*hfq E. coli* carrying empty vector, *ppk* or *hfq* expression vectors at N-48. **(H)** Recovery growth of N-24 Δ*ppk* and Δ*hfq E. coli* carrying *ppk* or *hfq* expression vectors after dilution into fresh Gutnick medium supplemented with 3 mM NH_4_Cl. Error bars indicate SD (*n* = 3). One-way ANOVA with was used in panels A and C; *p < 0.05, **p < 0.01, ***p < 0.001, ****p < 0.0001. **(I)** Subcellular localization of Hfq-mCherry in N-24 *hfq::hfq-mCherry* Δ*ppk* MG1655 carrying empty vector or overexpressing either *ppk* or *hfq*. Scale bars: 5 µm. All underlying data can be found in S2 Data.

To investigate how polyP affects the subcellular distribution of Hfq during N-starvation, we measured the relative diffusion rates and localization of the various Hfq subpopulations using single-molecule super-resolution microscopy (see Methods for details). We classified Hfq-PAmCherry trajectories by their overlap with Hfq foci and subsequently assigned the relative quantities of Hfq diffusive populations into three states: inclusion in a slow-diffusing condensate C-state, slow-moving non-condensate B-state or fast-diffusing free F-state (S2 Fig). Because Hfq has been previously identified as a nucleoid-associated protein [20–22], we assign the slow-moving B-state as nucleoid-bound; the Hfq molecules included in the C-state may also be nucleoid-bound. Consistent with the bulk observations noted above and fully supported by recently published results [23], we found that 47% of all Hfq molecules coalesce into one distinct, slow-diffusing C-state (i.e., the Hfq focus) in N-24 WT *E. coli* (Fig 2A) compared to only ~27% of Hfq molecules in the N-24 *ppk* deletion strain. Furthermore, we noted that in WT bacteria, the Hfq molecules that form the slow-diffusing C-state are balanced by a reduction in the pool of highly mobile “free” (F) Hfq molecules (~23%) and, to a lesser extent (~14%), in the pool of nucleoid-bound (B) Hfq molecules (Fig 2A). In the absence of polyP, however, the increase in Hfq in the condensate is primarily due to a reduction in the free pool, thus leaving close to 50% of non-focus-associated Hfq molecules bound to the nucleoid. These results agree well with previous in vitro findings, which showed that, at sufficiently high concentrations, polyP competes with DNA for Hfq binding [22]. The results furthermore suggested that polyP might be involved in increasing the concentration of cytosolic Hfq molecules that can be recruited to the focus by releasing Hfq molecules from the nucleoid. To next test whether polyP also affects the reverse process (i.e., focus dispersal), we replenished N-24 WT and Δ*ppk* strains with a fresh N source, which causes the dissolution of Hfq foci within a few hours [14] (Fig 2B and 2C). We observed that the Hfq foci disappear from WT *E. coli* at a rate that coincides with the decline in cellular polyP levels (shown in Fig 2D). We observed that the Hfq foci disappear from WT *E. coli* at a rate that appears to coincide with the decline in cellular polyP levels (shown in Fig 2D). Moreover, Hfq foci formed in the Δ*ppk* strain disappeared significantly faster than in WT *E. coli*, consistent with their observed differences stability upon hexanediol treatment (Fig 2B and 2C). In contrast, foci that formed in a mutant that lacks the *E. coli* polyphosphatase Ppx—and hence contains higher polyP levels during stress and shows a slower polyP decline upon N-replenishment compared to WT *E. coli* (Fig 2D)—revealed a significant delay in the disappearance of Hfq foci (Fig 2B and 2C). For example, while less than 40% of WT and 0% of Δ*ppk E. coli* contained any clearly visible Hfq foci after 6 h of N-replenishment, over 80% of Δ*ppx* bacteria still contained discrete Hfq foci (Fig 2C). These results supported our conclusion that changes in the cellular levels of polyP affect the formation as well as the dispersal of Hfq foci presumably by partitioning Hfq molecules across various cellular regions (cytoplasm, condensate and nucleoid).

### PolyP-mediated Hfq-foci formation correlates with improved stress resistance

One of the most crucial questions in the field of biological condensates concerns their physiological function(s). Previous studies revealed that the deletion of *hfq*, while not affecting *E. coli* growth under exponential conditions (S1A Fig), significantly reduces bacterial survival during N-starvation (recapitulated in Fig 2E) [14] and delays the restart of bacterial growth upon N-replenishment (Fig 2F). Yet, it had been unclear whether these phenotypes are connected to the lack of free Hfq, which functions as RNA chaperone and/or nucleoid-associated Hfq, which acts as suppressor of gene expression, or are due to the absence of foci-associated Hfq species. Based on our observation that the N-24 *ppk* deletion strain accumulates almost twice as much total Hfq compared to the N-24 WT strain (S1C Fig), the vast majority of which in either the free or nucleoid-bound form (Fig 2A), we now reasoned that the phenotypic analysis of the Δ*ppk* strain should shed light into the physiological role of Hfq foci during N-starvation. Analysis of N-starvation induced cell death revealed that while the deletion of *ppk* causes a slightly weaker phenotype compared to the phenotype of the Δ*hfq* strain (Fig 2E), both strains show an equally pronounced delay in the recovery upon N-replenishment (Fig 2F). Moreover, and even more importantly, we found that the combined deletion of both *ppk* and *hfq* genes phenocopied the growth defects of the *hfq* deletion alone (Fig 2E and 2F), implying that polyP and Hfq are likely part of the same pathway that protects *E. coli* against N-starvation stress. Rescue of the phenotypes by plasmid-borne expression of *ppk* or *hfq* confirmed that the observed growth defects are mediated by the respective gene deletions and not caused by any polar effects (Fig 2G and 2H). Importantly, overexpression of *ppk* in the absence of Hfq was insufficient to restore the growth defects, indicating that the growth defects are mediated primarily by Hfq. Yet overexpression of Hfq was able to fully rescue the phenotypes of the Δ*ppk* strain (Fig 2G and 2H) and, fully consistent with this activity, restored WT levels of Hfq foci formation in the Δ*ppk* strain (Fig 2I). Based on these findings, we now propose that the observed stress-induced accumulation of polyP acts on Hfq foci formation by reducing the critical concentration of Hfq for phase separation (i.e., the saturation concentration, *c*_sat_). This model would explain why upregulation of Hfq levels is not necessary for its phase separation and can take place even under stress conditions that inhibit de novo protein synthesis. Moreover, it would imply that the observed upregulation of Hfq levels in the *ppk* deletion strain might serve as a compensatory mechanism to partially offset the requirement for polyP for phase separation and allow for some foci formation even in the absence of polyP.

### PolyP is a hitherto unknown component of H-bodies

**Fig 3:**
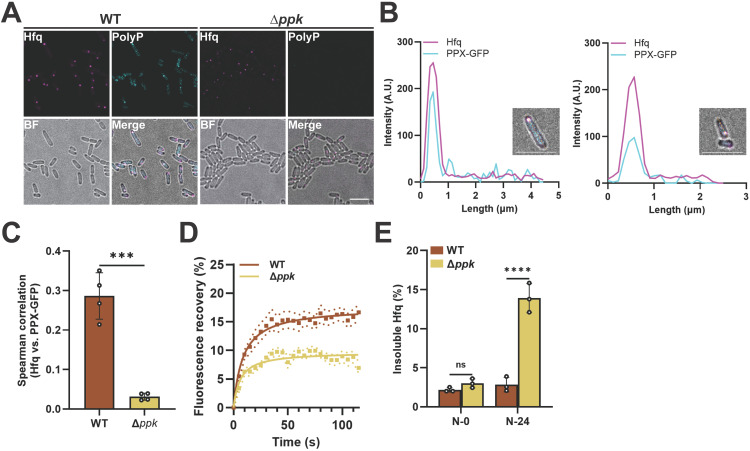
PolyP is a hitherto unknown component of H-bodies. **(A)** Co-localization of polyP and Hfq-mCherry in fixed N-24 WT (MG1655) and Δ*ppk hfq::hfq-mCherry*. PolyP is visualized by immunofluorescence using a -PPXPD-GFP probe while Hfq is visualized with endogenous mCherry fluorescence. **(B)** Plot profiles of Hfq-mCherry and PPXPD-GFP in select cells **(A)**, showing colocalization of Hfq and polyP. **(C)** Comparison of Spearman correlations between Hfq-mCherry and Ppx-GFP for nitrogen-starved cells of the indicated genotypes, demonstrating strong enrichment of polyP in the Hfq foci of WT cells. Comparison was made by unpaired *t* test, error bars show SD. ***p < 0.001 **(D)** Analysis of in vivo FRAP measurements using similarly sized Hfq-mCherry foci at N-24 in *hfq::hfq-mCherry* WT (MG1655) and Δ*ppk* cells. Ten cells were analyzed per sample. FRAP traces were fitted against a one-phase association curve: WT _t1/2_ = 8.8 s (95% CI 6.0 to 12.5 s), Δ*ppk*_t1/2_ = 6.2 s (95% CI 3.9 to 9.2 s), with solid lines representing fitted FRAP curves, square dots average fluorescence intensities at indicated time points and round dotted lines indicating the SEM. **(E)** Proportion of insoluble Hfq-mCherry at N-0 and N-24 in WT (MG1655) and *ppk* deletion strain as determined by quantitative western blot using antibodies against mCherry (*n* = 3, error bars indicate SD, ****p < 0.0001). Scale bars: 5 µm. All underlying data can be found in S3 Data.

Our results provided strong evidence for the conclusion that the stress-induced increase in polyP levels contributes to the phase separation of Hfq in vivo. To test whether polyP constitutes an integral component of Hfq foci, we fixed N-24 Hfq-mCherry expressing WT and Δ*ppk* strains and co-stained the cells with an antibody against mCherry to detect Hfq and a GFP-fused probe derived from the C-terminal polyP-binding domain of *E. coli* Ppx (PPXPD-GFP) [35] to visualize polyP (Fig 3A). We found a modest but significant colocalization of the two components in N-24 WT bacteria (Fig 3B and 3C), implying that polyP is a previously unrecognized component of stress-induced Hfq foci. Of note, however, only a subset of Hfq foci showed strong polyP enrichment. This result could be explained by inefficient permeability of the PPXPD-GFP probe into some Hfq foci or indicate that some Hfq foci form independently of polyP, consistent with our previous observations (Fig 1B). It is also important to mention that we observed a diffuse polyP signal in the cytoplasm/nucleoid region of the cells but did not observe any discrete polyP foci that were not associated with Hfq foci (Fig 3A and 3B). This polyP population might be associated with nucleoid-bound or free-diffusing Hfq (Fig 2A), or other charged proteins that might interact with polyP. To determine whether the presence of polyP affects the in vivo fluidic properties of Hfq condensates, we next compared the rate and extent of fluorescence recovery after photobleaching (FRAP), targeting similarly sized Hfq foci in both WT and Δ*ppk* strains (S3 Fig). We noted that while the kinetics of the Hfq-mCherry fluorescence recovery were similar and proceeded on the order of tens of seconds, the relative extent of recovery was significantly lower for Hfq-mCherry foci that formed in the absence of polyP compared to those formed in WT *E. coli* (Fig 3D). Subsequent Hfq solubility fractionation revealed that over 15% of Hfq molecules in the N-24 Δ*ppk* strain were insoluble compared to less than 5% of Hfq molecules in the N-24 WT strain (Fig 3E). This increased insolubility might contribute to a decrease in Hfq solubility within the foci or within the dilute phase, potentially affecting the diffusion of Hfq across the phase boundaries. In either case, these results suggested that polyP affects the fluidic properties of Hfq condensates.

### PolyP—A modulator of phase separating Hfq-RNA complexes

**Fig 4:**
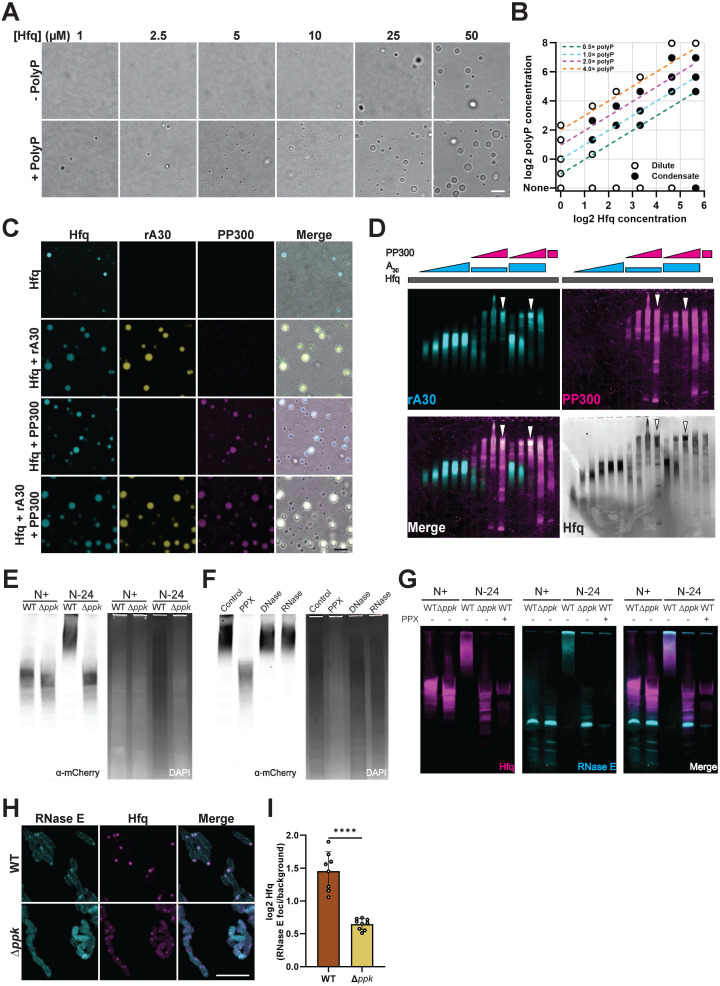
PolyP promotes Hfq (±RNA) condensate formation in vitro. **(A)** Brightfield images of Hfq condensates reconstituted with the indicated Hfq concentrations in the presence or absence of 1× polyP-300 (in Pi units). Samples were prepared in 50 mM NaCl and 20 mM HEPES pH 7.0. **(B)** Phase diagram of Hfq and polyP-300 in the same buffer as (A). Formation of condensates was computationally determined by quantitative image analysis as detailed in the Methods section. Lines corresponding with specific polyP:Hfq ratios are highlighted. **(C)** Fluorescence and brightfield images of condensates reconstituted with 50 µM Hfq (supplemented with 4% Cy3-Hfq S65C) in the presence or absence of 1 µM FAM-labeled rA_30_ or 100 µM AF647-labeled polyP-300. Samples were prepared in the same buffer as (A). **(D)** Electrophoretic mobility shift assays (EMSA). Lanes 1–6: 25 µM Hfq with 0, 0.5, 1, 2, 3, 4 µM FAM-rA_30_. Lanes 7–10 and lanes 11–14: 25 µM Hfq with 1 µM or 4 µM FAM-rA_30_, respectively, +25, 62.5, 125, 250 µM AF647-polyP300. Lane 15: 25 µM Hfq + 125 µM AF647-polyP300. A representative gel (*n* = 2) is shown. Hfq was stained with Coomassie blue. Hfq-polyP-RNA complexes indicated by vertical arrowheads. **(E)** Left panel: Native western blot of bacterial lysates from MG1655 *hfq::hfq-mCherry* WT and Δ*ppk* at N+ and N-24 using antibodies against mCherry. Right panel: UV-bleached DAPI stain of native gel shown in left panel. PolyP is shown as dark areas. **(F)** Left panel: Native western blot of cell lysates from N-24 MG1655 *hfq::hfq-mCherry* WT (MG1655) using antibodies against mCherry. Lysates were treated with the indicated enzymes for 2 h prior to electrophoresis. Right panel: UV-bleached DAPI stain of native gel as shown in left panel. **(G)** Native western blot of N+ and N-24 lysates from *hfq::hfq-mCherry rne::rne-mTQ2* WT (MG1655) relative to Δ*ppk* using antibodies against mCherry (Hfq) and GFP (RNase E). N-24 samples were left untreated (−) or digested with Ppx for 2 h (+) prior to electrophoresis. **(H)** Subcellular localization of Hfq-mCherry and RNase E-mTQ2 in N24 WT (MG1655) or Δ*ppk*. Scale bars: 5 µm. **(I)** Distribution of log2 ratios of RNase E intensities in Hfq foci (see Methods for details) vs. cellular background. Each data point shows a separate imaging field taken across two separate biological replicates. Scale bars: 5 µm. All underlying data can be found in S4 Data.

To investigate the interaction between Hfq and polyP in more detail, we purified Hfq and analyzed its phase separation properties in the absence and presence of polyP-300. Under our buffer conditions (i.e., HEPES buffer, 50 mM NaCl, no crowding reagents), Hfq forms very few if any detectable droplets at protein concentrations below 25 µM but reproducibly phase separates at higher protein concentrations (Fig 4A). In the presence of polyP, droplet formation was significantly increased and clearly detectable at Hfq concentrations as low as 1 µM. A phase diagram revealed that while molar ratios of polyP (in Pi units) to Hfq of 2:1–3:1 promote droplet formation, further increase in the polyP concentration causes the re-entry of Hfq droplets into the solute phase (Fig 4B). Co-incubation of fluorescently labeled Hfq and polyP confirmed that the two biomolecules co-partition into the droplets (Fig 4C). Inspired by previous work, which demonstrated that Hfq binds polyadenylated mRNAs and helps to prevent their degradation by the RNA degradosome in vitro [36], we next wondered whether the presence of polyP would affect RNA-Hfq interactions within the condensates. Therefore, we differentially fluorophore-labeled Hfq, polyP and rA_30_ RNA (a known interactor of Hfq [37]), and co-incubated the three components. While the addition of either polyanion increased the relative number of droplets that Hfq forms at any given concentration and co-localized with Hfq, their combined presence further enhanced in vitro droplet formation and led to the formation of ternary condensates (Fig 4C). These results implied that in contrast to DNA binding, which appears to be mutually exclusive with polyP binding [22], Hfq simultaneously binds to both rA_30_ RNA and polyP-300. We independently confirmed this simultaneous interaction by using an electrophoretic shift assay (EMSA), which demonstrated that addition of increasing amounts of polyP-300 to discretely migrating pre-formed Hfq-rA_30_ RNA complexes causes the formation of ternary complexes that stain positive for all three components (Fig 4D). The migration pattern of the ternary complexes was similar to the ladder-like migration of Hfq-polyP complexes (Fig 4D, last lane), suggesting that polyP-300 scaffolds multiple rA_30_ RNA-binding Hfq hexamers into larger oligomers that phase separate in vitro and potentially also in vivo.

### PolyP mediates higher oligomerization of Hfq under N-starvation in vivo

To test whether the observed in vivo Hfq foci formation also involves the polyP-dependent formation of HMW Hfq assemblies, we prepared lysates from Hfq-mCherry expressing WT and Δ*ppk* bacteria grown under N-0 and N-24 conditions, and analyzed the migration of Hfq-mCherry on native western blots. As expected, Hfq-mCherry migrated as a low molecular weight (LMW) species in lysates prepared from either set of N-0 bacteria. In contrast, the majority of Hfq-mCherry in N-24 WT lysates migrated in the form of HMW assemblies (Fig 4E), reminiscent of in vitro formed complexes between Hfq, polyP, and rA30 (Fig 4D). In the N-24 Δ*ppk* lysate, however, most of the Hfq-mCherry signal remained in the LMW region, suggesting that the smaller Hfq foci that form in the Δ*ppk* strain are unstable and dissociate during sample preparation. Incubation of N-24 WT lysates with the polyP-degrading yeast polyphosphatase (yPpx) [38]—but not with DNase I nor RNase A—prior to the native PAGE analysis caused the dissociation of the majority of HMW complexes into LMW Hfq species (Fig 4F), further supporting the idea that polyP serves as an in vivo scaffold for HMW complex formation of Hfq. To test whether the HMW complexes of Hfq that we observed in N-24 WT lysates correspond to foci-associated Hfq, we next analyzed the migration behavior of RNase E, a known component of N-starvation-induced Hfq foci [13] (see below). For these experiments, we tagged RNase E with mTurquoise2 (mTQ2) at its endogenous loci, expressed it in the Hfq-mCherry expressing WT or Δ*ppk* strains and prepared lysates from N-0 and N-24 cells. As shown in Fig 4G, we found that part of the RNase E-mTQ2 signal associates with the HMW species of Hfq in N-24 WT lysates but not in the Δ*ppk* strain or in Ppx-treated WT lysates. Live-cell fluorescent imaging of WT and Δ*ppk* strains co-expressing RNase E-mNG and Hfq-mCherry confirmed our native PAGE results and demonstrated that upon N-starvation, RNase E forms membrane-enriched foci that colocalize with Hfq foci to a significantly higher extent in WT *E. coli* compared to the Δ*ppk* strain (Fig 4H and 4I). These results collectively suggest a potential mechanism by which polyP promotes Hfq foci formation. While Hfq hexamers have the intrinsic propensity to form smaller oligomers that can further phase separate into condensates, polyP facilitates this process by scaffolding Hfq hexamers into larger oligomers, which favor phase separation due to their lower mixing entropy and stronger attractive interactions [39].

### H-bodies—Control centers for protein translation and RNA processing

**Fig 5:**
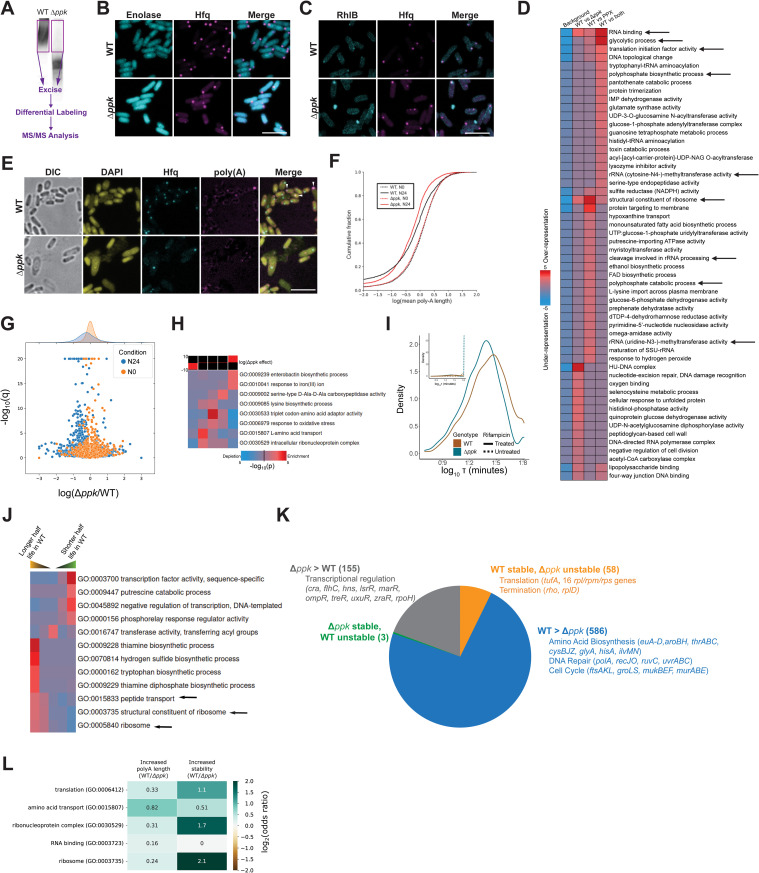
Compositional and functional properties of Hfq/polyP-foci. **(A)** Diagram showing the workflow of MS/MS on native gel-resolved samples. Excised regions are indicated by purple rectangles. **(B)** Subcellular localization of Hfq-mCherry and Eno-mTQ2 in N24 WT (MG1655) or Δ*ppk*. Scale bars: 5 µm. **(C)** Subcellular localization of Hfq-mCherry and RhlB-mTQ2 in N24 WT (MG1655) or Δ*ppk*. **(D)** GO term analysis using iPAGE (see Methods for details), indicating GO terms enriched by more than 2-fold in WT vs. Δ*ppk* pulldowns, untreated vs. yPpx-treated pulldowns, or both. In each case, red boxes indicate over-representation of genes annotated with a particular GO term in a given group. **(E)** Colocalization of poly(A) RNA and Hfq as shown by fluorescent in situ hybridization against poly(A) RNA in *hfq::hfq-mCherry* WT (MG1655) and *Δppk* at N24. **(F)** Cumulative distributions of estimated mean untemplated poly(A) lengths across the entire transcriptome in each of the indicated conditions. **(G)** Changes in poly(A) tail length (x-axis) and statistical significance (y-axis) comparing *Δppk* and WT cells under each of the indicated conditions; the leftward shift in *Δppk* cells specifically at the N24 time point is clearly apparent. **(H)** GO term enrichments and depletions among the indicated quintiles of changes in poly(A) tail length upon deletion of *ppk*; terms in the lower quintiles show relative decreases in poly(A) tail length upon *ppk* deletion. **(I)** Distributions of the observed transcript half-lives in rifampicin treated *hfq::hfq-mCherry* WT (MG1655) and *Δppk* (S8 Data). Decay constants outside of the range 5-65 min are clamped for plotting purposes. Inset: Comparison of untreated and treated distributions. **(J)** GO term enrichments and depletions among the indicated quintiles of changes in transcript half-life upon deletion of *ppk*. **(K)** Key GO terms and representative genes enriched in each of four major categories of *ppk* induced stability changes (stable vs. unstable: *τ* in one genotype >65 min and <55 min in the other; WT > *Δppk*/ *Δppk* > WT: *τ* in both genotypes <65 min but t in one genotype increased by ≥10 min) along with representative genes. **(L)** Enrichments of genes in each indicated GO term among genes showing higher poly(A) tail lengths or stability in WT cells relative to *Δppk* cells in the N-24 condition, shown as log2-scaled odds ratios (small sample method plus Yates’ continuity correction, calculated using the epitools R package). All underlying data can be found in S5 Data.

Based on these results, we now reasoned that the proteomic analysis of the gel region encompassing the HMW complexes of Hfq in WT lysates and its comparison to the same gel region of the Δ*ppk* lysates might provide an opportunity to characterize the protein composition of Hfq foci without the need of developing techniques to isolate the foci, an often extremely challenging endeavor [40]. We thus excised the respective gel regions and conducted comparative tandem mass spectrometry (MS/MS) analysis (Fig 5A). We obtained highly reproducible results and identified 76 proteins which are significantly enriched in the gel region corresponding to the HMW Hfq complexes of N-24 WT lysates compared to the corresponding gel region of the Δ*ppk* lysate (S1 Table, full results on all detected proteins are in S7 Data). Importantly, and, very similar to our results with Hfq and RNase E (see above), we found that the treatment of the WT lysates with yPpx but not RNase A depleted many of these proteins from the HMW region of the native PAGE (S1 Table), implying that the identified proteins are indeed associated with Hfq through the action of polyP and the observed enrichment hence unlikely caused by relative differences in the expression levels of these proteins in WT *versus* the *ppk* deletion strain. Moreover, among the list of proteins that we found specifically associated with the HMW complexes of Hfq were two proteins which had previously been shown to colocalize with Hfq foci under N-starvation: enolase (Eno) and the RNA helicase RhlB. In an effort to further validate our results, we endogenously mTQ2-tagged Eno or RhlB in Hfq-mCherry expressing WT and *ppk* deletion strains and monitored their N-starvation-induced foci formation and co-localization with Hfq. While we observed a mostly diffuse localization of Eno-mTQ2 that made focus calling impossible (Fig 5B), we confirmed that RhlB-mTQ2 foci strongly coincided with Hfq-mCherry in the presence of polyP but not in its absence (Fig 5C). We note, however, that proteins forming higher oligomers in the presence of polyP but not directly interacting with Hfq could also be identified via the gel extraction method described above. To eliminate these, potentially false positive candidates, we co-immunoprecipitated Hfq from N-0 and N-24 WT and Δ*ppk* lysates and focused our analysis on those proteins that we identified in our HMW complexes. Of the 76 proteins that we identified to co-migrate with Hfq in a polyP-dependent manner on native PAGE, 45% were found quantitatively also in these pulldowns (excluding the mCherry-only, non-Hfq-tagged control; see S8 Data). This result was despite us only detecting ~8% of the proteome in the pulldowns, making it a 5.7-fold enrichment over what we would expect by chance, and hence statistically highly significant (*p* < 10^−15^, proportion test). These results further increased our confidence that the proteins that co-migrate with Hfq in the native PAGE of WT lysates but not the Δ*ppk* lysates (or yPpx-treated WT lysates) are likely proteins that are sequestered into Hfq foci in a polyP-dependent manner and encouraged a deeper analysis of the identified HMW Hfq-associated proteins through gene ontology (GO) enrichment analysis. We found that this set of proteins revealed a strong over-representation for proteins associated with ribonucleoprotein complex assembly, ribosome assembly, and RNA modifications (Fig 5D). Of particular interest were 23 translation-associated proteins that showed a log2-fold enrichment in WT relative to Δ*ppk* cells between +1.1 to +6.6 and included all three translation initiation factors (IFs), several ATP-dependent RNA helicases, numerous rRNA modifying enzymes and ribosomal subunits (S1 Table). Based on these findings, we concluded that polyP affects the protein composition of Hfq foci, which appear to be organizing centers for proteins involved in protein translation, RNA processing and decay.

### H-bodies regulate transcript stability during N-starvation

Based on previous reports on Hfq’s role in stabilizing poly(A)-mRNAs against degradation by the RNA degradosome [36] and our finding that Hfq foci contain proteins involved in RNA processing, we now wondered whether H-bodies sequester poly(A)-transcripts. To test for the presence of poly(A) transcripts in Hfq foci, we conducted fluorescence in situ hybridization (FISH) experiments in N-24 WT and Δ*ppk* strains. Although we observed clear co-localization between poly(A)-transcripts and Hfq foci in about 20% of N-24 WT cells per field of view and no such co-localization in any Δ*ppk* cells, the low signal to noise ratio of the polyA probe made quantification of these data challenging (Fig 5E). As an alternative approach, we therefore tracked the lengths of untemplated poly(A) sequences present at the 3′ end of transcripts in both WT and Δ*ppk* cells at the N-0 and N-24 conditions (see Methods for details). At N-0, absence of *ppk* had no discernible systematic effect on the poly(A) tail lengths of the transcripts. As cells entered deep N-starvation conditions, we observed a general decrease in the mean poly(A) tail lengths which was significantly more pronounced in cells lacking polyP (Fig 5F and 5G). Given that polyadenylation of *E. coli* mRNA classically leads to reduced transcript stability [41], these results suggested that under N-starvation conditions, H-body formation might aid in sequestering mRNAs with longer poly(A) tails which would otherwise be degraded by the degradosome [42]. Of particular interest was the finding that many of the genes with increased poly(A) tail lengths in the WT cells compared to the Δ*ppk* cells encode for proteins involved in amino acid uptake and the translational machinery (Fig 5H). These results offered a functional connection to the classes of proteins observed to be enriched in Hfq/polyP-foci (S1 Table) and suggested the possibility of a concerted regulation and stabilization of those genes by Hfq foci formation at both the transcript and protein levels.

Based on these results, we now wondered whether the observed changes in poly(A) tail lengths might serve to differentially stabilize select mRNA transcripts during N-starvation. To directly monitor transcript stability, we added the transcriptional inhibitor rifampicin and conducted transcriptional shutdown experiments in N-24 WT and Δ*ppk* cells over a 60 min time course, using spike-ins for normalization [43] (see Methods for important details on the experimental design and interpretation). In both WT and Δ*ppk* cells, we observed clearly normal distributions (Fig 5I; S9 Data), yet with a significant shift toward shorter half-lives for transcripts in the Δ*ppk* cells relative to WT (~14% decrease in median half-life; p < 10^-15^, Wilcoxon rank sum test). We calculated that about 33% of all transcripts with identifiable half lives in each condition were substantially less and only about 8% transcripts substantially more stable in N-starved Δ*ppk versus* WT bacteria (S4A Fig). Gene set enrichment analysis revealed several categories of genes for which transcript stability was altered by deletion of *ppk* during N-starvation, including systematic stabilization of transcripts encoding genes involved in transcriptional regulation, and broad destabilization of transcripts encoding genes involved in cell division, amino acid metabolism, and protein translation (Fig 5J). Deeper analysis of specific genes of interest among the transcripts that are highly stable in WT during N-starvation but significantly destabilized in the Δ*ppk* bacteria showed a particular enrichment for several components of the translational machinery (i.e., 30S small and large ribosomal subunits) (Figs 5K and S4B). These results, and particularly the strong *ppk-*dependent stabilization of transcripts encoding core translational proteins, suggested that polyP-dependent Hfq foci formation plays a role in preserving transcripts that are necessary to rapidly restart protein synthesis and growth while targeting potentially less-essential transcripts for turnover. It is especially notable that the transcripts encoding Hfq/polyP foci components (as identified by MS on the HMW complexes; see above) showed a median 28% larger increase in half-life (and thus higher stability) in the presence of polyP, relative to other transcripts (*p* = 5.2*10^−5^, Wilcoxon rank sum test), reflecting a concerted preservation of the proteins involved in Hfq foci across all stages of synthesis and degradation. Finally, by cross-referencing our data on poly(A) tail length and transcript stability, we revealed that the transcripts involved in the core polyP-preserved processes such as translation are indeed among the ones with increased poly(A) tail lengths and increased stability in WT compared to the Δ*ppk* bacteria (compare Fig 5J and 5H). Genes contained in the GO terms identified by this analysis generally showed an enrichment for both increased poly(A) tail lengths and increased stability in WT N-24 cells relative to Δ*ppk* bacteria under the same condition (Fig 5L). In summary, these results imply that the polyP-dependent sequestration of transcripts into Hfq-foci preserves their poly(A) tails, thereby contributing to their stabilization during N-starvation.

### PolyP plays a critical organizational role in eukaryotic P-body formation

**Fig 6:**
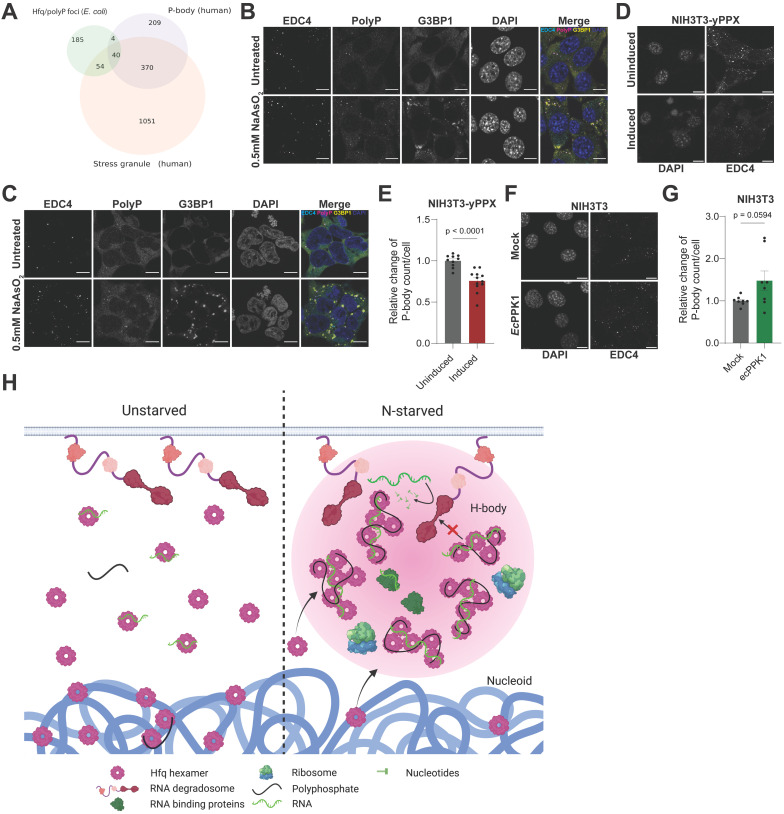
PolyP supports P-body formation in mammalian cells. **(A)** Euler diagram showing the overlaps of GO terms annotated to proteins identified in the Hfq/polyP foci vs. those in human P-bodies and stress granules (S7 Data). **(B)** NIH3T3 and **(C)** HEK293 cells, either untreated or treated with 0.5 mM sodium arsenite (NaAsO_2_) for 30 min were fixed and stained for Processing (P)- bodies using antibodies against the marker protein EDC4 (cyan), stress granules (SG) using antibodies against the marker protein G3BP1 (yellow), polyP using the PPXBD-mcherry protein (magenta) and DAPI to visualize the nucleus (blue). A representative experiment is shown (*n* = 3). **(D)** A stable NIH3T3 cell line expressing an inducible yPpx was fixed after 24h of addition of the inducer shield and stained for the P-body marker EDC4. **(E)** Quantification of the EDC4 positive punctate structures shown in (D). The average number of P-bodies counted in the uninduced (D) or untransfected (F) cells was normalized to 1. Image analysis was automated via thresholding to get an unbiased count of P-bodies. An unpaired *t* test was used to assess statistical significance (*n* = 3 biological replicates; data points represent all imaging fields pooled across those experiments). **(F)** NIH3T3 cells transiently transfected with the *E. coli* Ppk were fixed after 24 h of transfection and stained for the P-body marker EDC4. **(G)** Quantification of the EDC4 positive punctate structures shown in (F). Each data point represents the average P-body count per cell in an image of at least 20 cells. The average number of P-bodies counted in the uninduced (D) or untransfected (F) cells was normalized to 1. Image analysis was automated via thresholding to get an unbiased count of P-bodies. An unpaired *t* test was used to assess statistical significance (*n* = 3 biological replicates; data points represent all imaging fields pooled across those experiments). Scale bars: 10 µm. **(H)** Current working model of H-body formation and function. Upon N-starvation, *E. coli* accumulates long-chain polyP, which scaffolds Hfq-hexamers into higher oligomers, giving rise to phase separated condensates. PolyP promotes the selective sequestration of poly-adenylated RNAs and RNA-binding proteins into H-bodies and facilitates their association with the membrane-tethered RNA degradosome. H-body formation confers specific protection of transcripts involved in translation and metabolism while facilitating rapid turnover of transcripts that are likely less critical for survival. Figure panel was created with *BioRender, Oleson,* B. *(2026)* *https://BioRender.com/lf1mrtp.* All underlying data can be found in S6 Data.

At the functional level, we cross-referenced the GO terms with which Hfq/polyP focus associated proteins were annotated, and compared them with the corresponding lists of GO terms for proteins associated with mammalian Processing (P-) bodies, which are involved in regulating RNA turnover, and SGs, which sequester the translational apparatus [44]. Our analysis revealed that 35% of the Hfq/polyP focus associated GO terms overlapped with SG/P-body GO terms (Fig 6A; S7 Data) [12]. This enrichment was significantly higher than what would be expected by chance (*p* = 0.004, 0.001, and 0004 for the P-body, SG, and joint overlaps, respectively, via resampling tests). These results raised the question whether polyP, which is abundantly present also in mammalian cells [45,46], might be equally involved in eukaryotic condensate formation. We tested two mammalian cell lines, NIH3T3 (Fig 6B) and HEK293 (Fig 6C) and stained them with the P-body-specific marker protein EDC4 [47] or the SG-specific marker protein G3BP1 [48]. As previously noted, both cell lines contain some visible P-bodies but no distinct SGs under non-stress conditions [49,50] and reveal an increase in size and number of both types of condensates upon treatment with arsenite [51,52]. When we co-stained these cells before and after arsenite treatment with the mCherry tagged polyP-binding domain, we found a clear co-localization between polyP and EDC4 but not between polyP and G3BP1 (Fig 6B and 6C). We did observe some clearly visible polyP-positive P-bodies in close vicinity to SGs; however, these condensates have been observed before and suggested to be involved in exchanging select proteins and substrates between the different condensates [53,54]. These results strongly suggested that polyP specifically interacts with components of P-bodies but not SGs. To test whether manipulation of cellular polyP levels affects the formation of P-bodies, we constructed two NIH3T3-based cell lines; we generated stably transfected NIH3T3 cells expressing an inducible version of the yPpx, which significantly decreased cytosolic polyP levels within 24 h of induction (S5A, S5B), or transiently transfected NIH3T3 cells with a vector encoding *E. coli* Ppk, which significantly elevated the levels of polyP compared to non-transfected control cells (S5C, S5D). Indeed, and similar to our results *in E. coli*, where depletion of polyP decreases and elevated levels of polyP increases Hfq foci formation, we observed a clear relationship between polyP levels and P-body formation in mammalian cells. Cells containing lower levels of polyP showed a significant drop in P-body numbers (Fig 6D and 6E) whereas cells with higher-than-normal levels of polyP had increased P-body counts compared to non-transfected cells (Fig 6F and 6G). These results suggest that polyP contributes to—and possibly even also drives—P-body formation in the context of mammalian cells, leading us to conclude that polyP’s role in condensate formation has persisted across evolution even if the precise condensates being studied in the systems here may have arisen independently.

## Discussion

After more than a decade of extensive research, biomolecular condensates have been recognized as critical subcellular organizing principles, particularly relevant as rapid response mechanisms to environmental stressors. In the cytosol of mammalian cells, formation of P-bodies and SGs has been shown to contribute to regulating mRNA turnover, translational repression, and the sequestration of stalled components of the translational machinery during stress, promoting a quick recovery once the stress conditions are over [55]. In bacteria, the condensates that have been studied in depth to date seem equally associated with stress response and rapid growth recovery [56–58]. Yet, the common principles of what leads to the sudden phase separation of select proteins under these stress conditions remain unclear. In vitro studies demonstrated the strict reliance of condensate formation on the concentration of protein(s) as well as the presence of polyanions, such as RNAs, which promote multivalent interactions, thereby reducing the *c*_sat_ of the proteins involved [26]. However, most known stress conditions that cause protein condensate formation stall de novo protein synthesis and do not produce excess levels of RNA [7,59,60], raising the question what other stress-responsive mechanisms might be in play that trigger condensate formation. Here we demonstrate that polyP, an ancient and ubiquitous inorganic biopolymer, whose synthesis and degradation are dynamically regulated in response to stress [30,61], supports the formation and functional regulation of stress-induced condensates in both prokaryotes and eukaryotes. We discovered that in *E. coli*, the accumulation of long-chain polyP—either naturally produced in response to N-starvation or osmotic stress, or artificially induced by the expression of an “overactive” polyphosphate kinase (i.e., Ppk10k) [34]—significantly increases the fraction of bacteria with a single polar-localized and functional Hfq focus, whereas loss of polyP production substantial reduces both the number of cells with Hfq foci, and the sizes/intensities of those foci. We found that polyP appears to play several interdependent roles in both the formation and the function of these condensates: (1) It contributes to the release of nucleoid-bound Hfq, thus increasing the concentration of free Hfq that is available to phase separate; this effect is likely due to the direct competition between polyP and DNA binding to Hfq [22]; (2) It scaffolds RNA binding-competent Hfq into higher oligomeric complexes, a feature commonly known to increase phase separating propensities [62]; (3) It stabilizes Hfq condensates as reflected by their higher FRAP recovery, higher hexanediol resistance, and longer half-life upon return to non-stress conditions; and (4) It pivots Hfq’s function towards the stabilization of polyadenylated transcripts essential for translation and cell division while facilitating the decay of those that appear to be less crucial for bacterial survival. These latter findings raise the obvious question as to how polyP might manage this challenging task. The structural similarity between polyP chains and the phosphate-rich backbone of RNA/DNA makes it reasonable to assume that polyP chains interact with one or more of the various nucleic binding surfaces of Hfq [22]. In fact, our previous studies, which focused on the role of polyP on nucleoid-associated Hfq, demonstrated that at low polyP concentrations (such as during exponential growth under N-replete conditions), polyP binding to Hfq changes the DNA-binding specificity of Hfq from GC-rich to AT-rich sequences [22]. We now posit that at higher polyP concentrations (such as found during N-starvation), polyP binding to other surfaces in Hfq (for instance the DNA binding proximal surface) might similarly alter the RNA binding specificity of Hfq. It is reasonable to assume that polyP binding might compete with low-affinity binding sequences and thus, by default, select for binding to the highest-affinity sequences, such as transcripts with long poly(A) tails. This feature would allow cells to control the sequence specificity of multi-functional nucleic acid binding-proteins simply by changing the relative concentration of polyP. Inevitably, however, detailed structural studies are needed to test this model and dissect the precise mechanisms by which H-body formation leads to the selective binding of polyadenylated transcripts during select stress conditions.

Previous work revealed that polyadenylation of *E. coli* mRNA leads to reduced transcript stability [41], a result that was further supported by very recent reports, which demonstrated that the loss of the *E. coli* poly(A) polymerase PcnB leads to a pervasive transcript stabilization [63]. Similarly, transcript stabilization has also been observed upon the interaction of polyadenylated transcripts and Hfq [36], a finding that is largely consistent with our results. Yet, we argue that Hfq likely plays a more nuanced role, especially under stress conditions that lead to the formation of H-bodies. Under these stress conditions, we found that H-bodies preferentially stabilize transcripts with longer poly(A) tail lengths. We furthermore revealed that the transcripts that are enriched for longer poly(A) tails and stabilized in a polyP-dependent manner encode for proteins and cellular pathways that match those enriched in H-bodies at the protein level. Because both Hfq and polyP have been shown to antagonize RNase E-mediated degradation of polyadenylated mRNA in vitro [36,64], we now speculate that polyP-enriched Hfq foci sequester the RNA degradosome in an inactive form, thus serving as a hub for selective mRNA storage. It is important to note that the polyP-dependent selective sequestration of mRNA transcripts with longer poly(A) tails would also be consistent with N-starvation conditions that might cause a polyP-dependent global increase in polyadenylation. Yet, we consider this possibility unlikely because N-starvation appears to lead to an overall reduction in the average poly(A) tail lengths (Fig 5F). We also wondered whether the activity of PcnB, the enzyme responsible for the formation of poly(A) tails, might be altered. This idea was based on recent results, which showed that PcnB is inhibited at low cellular ATP concentrations [63]. However, although we did observe slightly lower ATP concentrations in N-24 *ppk* deletion bacteria compared to N-24 WT *E. coli*, the ATP concentrations of N-starved bacteria did not go below the ATP levels of N+ bacteria. These results make significant differences in PcnB activity due to changing ATP levels also an unlikely explanation for our observations.

Besides knowing how condensates form, another crucial question concerns their specific roles in biological processes. Our data show that the *ppk* deletion mutant, which has twice the levels of Hfq but is defective in the formation of proper Hfq foci, has growth defects that are highly similar to the growth defects of the *hfq* mutant and, even more relevant, not additive when combined with the deletion of *hfq*. Since we cannot fully exclude that the disruption of a major regulator such as *hfq* has effects on various aspects of cellular physiology that could change the needs and/or roles of polyP, suitable separation-of-function mutants of Hfq that bind polyP but do not phase separate and vice versa are needed. However, such separation of function mutants may prove impossible to generate, as we suspect that the ability of Hfq to bind polyP and phase separate may be essentially synonymous. Nevertheless, centered on these phenotypical results, we propose that the observed polyP-dependent transcript stabilization and degradosome/translational apparatus sequestration by Hfq foci is the mechanism that promotes improved N-starvation survival. We base this model on several lines of evidence, including our previous results showing that the RNA chaperone activity of Hfq is insensitive to the presence of polyP across a wide range of known client mRNA/sRNA pairs [22], making it unlikely that the beneficial effects of polyP accumulation under N-starvation are connected to altered mRNA/sRNA interactions. Moreover, the observed polyP-mediated dissociation of Hfq from the nucleoid under N-starvation should, if anything, make bacteria more (rather than less) sensitive to stress due to a potential increase in the transcription of prophage genes and mobile genetic elements [22]. Finally, the strong consistency of the gene classes that we observe being stabilized at both transcript and protein levels in Hfq foci that contain polyP is most simply explained by the presence of a system selectively targeting those pathways. Yet, future mechanistic dissections of the relative contributions of different binding surfaces and activities of Hfq to both polyP-dependent phase separation and survival of N-starvation are clearly needed to provide the necessary additional insights into this system and as template for understanding polyP-dependent phase separation in other biological contexts (both in bacteria and in eukaryotic cells).

Finally, our study suggests that polyP might play a potentially equivalent role as modulator for P-bodies, a stress-induced hub for RNA turnover in eukaryotes. We found that H-bodies share many similarities with cytosolic condensates found in many eukaryotic cells: mammalian P-bodies, like H-bodies, form around the Sm-domain containing heptameric protein Lsm1–7, a eukaryotic counterpart of Hfq; accumulate mRNAs with short poly(A) tails; and associate with components of the RNA degradation machinery to regulate turnover [12,65]. Mammalian SGs share with H-bodies the fact that they assemble specifically in response to stress conditions that trigger translation inhibition, and accumulate ribosomal subunits and translation IFs, indicative of stalled ribosomes [47,66,67]. Although considered separate entities, mammalian P-bodies and SGs are often found in close spatial proximity and share select proteins and RNAs [53]. Since bacterial H-bodies combine features of both types of condensates on both compositional and functional levels, we propose that they might represent some of the most ancient forms of functional LLPS-driven condensates. Given that polyP concentrations are highly sensitive to environmental conditions that lead to condensate formation, we now speculate that polyP might be the quintessential and, based on its prebiotic origin, the ancestral modulator of condition-dependent protein condensation in vivo. While recent studies revealed that select stress conditions induce the synthesis of mammalian polyP as well [35,68], much still has to be learnt about the system involved in the synthesis and regulation of eukaryotic polyP homeostasis. Nevertheless, our studies suggest that polyP, a simple and ancient biopolymer, potentially carries out a conserved scheme of stress response by triggering and functionally regulating biomolecular condensates in a wide range of organisms.

### Resource availability

**Lead contact:** Requests for further information and resources should be directed to and will be fulfilled by the lead contact, Ursula Jakob (ujakob@umich.edu).

**Materials available:** The strains and cell lines constructed for this paper are available from the lead contact without restriction.

### Methods

**Bacterial strains and plasmids.** All strains, plasmids and oligonucleotides used in this study were derived from *E. coli* strain MG1655 and are listed in S3 Table. Alleles were deleted or fluorescently labeled via *λ* Red recombination method [69]. Labeled alleles were transduced into various mutant strains using P1*vir* bacteriophages from appropriate donors. All plasmids used in this study were constructed with restriction digestion and ligation or Gibson assembly and verified by sequencing.

**Bacterial growth conditions.** WT and mutant bacteria were grown in Gutnick minimal medium (33.8 mM KH_2_PO_4_, 77.5 mM K_2_HPO_4_, 5.74 mM K_2_SO_4_, 0.41 mM MgSO_4_) containing 0.4% (w/v) glucose, 10 mM NH_4_Cl and M9 trace elements (“N+ medium”) [70]. For N-starvation experiments, overnight cultures in N+ medium were inoculated in Gutnick minimal medium containing 0.4% (w/v) glucose, 3 mM NH_4_Cl and M9 trace elements (“N- medium”). For osmotic stress experiments, cultures at mid-exponential phase in LB medium were spun down, resuspended in equal volume of LB medium containing 1.17 M NaCl, and let grow for 4 h. Under all conditions, bacteria were grown at 37 °C with 200 rpm shaking. During growth in N-medium, the ammonium level in the medium was determined by using the Ammonia/ammonium microplate assay kit following the manufacturer’s protocol (#orb545637, Biobyrt). When indicated, kanamycin (100 µg/ml), ampicillin (200 µg/ml) and/or chloramphenicol (50 µg/ml) were added. Optical density of the culture was measured on a UV/vis spectrophotometer. The number of viable cells in the culture were measured as CFU/ml after serial dilutions on LB agar plates and incubation overnight at 37 °C.

**Fluorescence microscopy and FRAP measurements.** Imaging of fluorescently labeled bacteria was performed on a Leica SP8 inverted microscope with 100× oil immersion objective, driven by LAS X software (Leica GmbH, Mannheim, Germany). Bacteria were grown under the conditions described above. At the indicated time points, 1 mL of cells were spun down, washed and resuspended in a small volume of Gutnick minimal medium without glucose or NH_4_Cl. Cells were loaded onto a cover slip and immobilized on 1% agarose pad made from the same medium [14]. For imaging of N+ cells, medium with 3 mM NH_4_Cl and 0.4% w/v glucose was used for pad preparation. For imaging of the nucleoid, cells were stained with 1 µg/mL 4′,6-diamidino-2-phenylindole (DAPI, #D1306, Thermo Fisher Scientific) before being spun down and washed. For quantification of the foci, a minimum of 100 cells per sample was analyzed. All foci quantifications were performed in a blinded manner with ImageJ. FRAP measurements were performed with the Zoom In mode, using the 405 and 587 nm lasers together at 100% intensity. Three pre-bleach images were acquired, followed by photobleaching and imaging every 5 s for 115 s. Fluorescence intensity was measured by LAS X software. Recovery data were plotted and fitted against a one-phase association curve with GraphPad Prism.

**Hfq focus area/intensity quantitation.** Cells were imaged as described above, acquiring DAPI, mCherry (Hfq), and brightfield channels at each time point. Cells were then segmented using omnipose [71] with the bact_fluor_omni model applied to the DAPI channel, using a mask threshold of −1 and flow threshold of 0. Each cell mask was then further expanded by four pixels using the skimage binary_dilation operator, and a minimal convex hull of the resulting points generated to define the final cell boundaries. A global robust threshold for the pixel-level Hfq intensity needed to constitute an Hfq focus was defined as being at least 3.5 times the median absolute deviation (MAD) above the observed median Hfq signal within cells. We then defined as focal centers all locations that were both local maxima in terms of Hfq signal, and which exceeded the global threshold defined above. Each region exceeding the Hfq threshold was then expanded to encompass all contiguous pixels (using 1-connectivity) that exceeded the threshold and labeled as a focus if it contained at least one potential focus center, and foci containing fewer than three pixels were discarded. We then calculated the areas and intensities of the thus-averaged foci, taking the mean of the indicated quantity across all imaging fields for each replicate separately.

**Bacterial immunofluorescence microscopy in fixed cells.** N-24 cultures of WT and Δ*ppk hfq::hfq-mCherry* strains were cultivated as described. Cells equivalent to 1 ml OD_600_ = 1.5 were collected and fixed with fresh 4% paraformaldehyde (#1578100, Electron Microscopy Sciences) for 15 min at room temperature followed by 30 min on ice. Cells were spun down and washed three times in phosphate-buffered saline (PBS), resuspended in 1 ml GTE buffer (50 mM glucose, 10 mM EDTA pH 8.0, 20 mM Tris-HCl pH 7.5) containing 20 µg/ml lysozyme, and incubated for 1 min. 20 µl of cells were loaded onto a poly-L-lysine (#P8920, Sigma-Aldrich) coated coverslip (#72,230–01, Electron Microscopy Sciences) for 15 min. Coverslips were washed three times with PBS, and dried at room temperature. After rehydrating with PBS, samples were blocked with 2% bovine serum albumin (#A3059, Sigma-Aldrich) in PBS for 20 min. A PPXBD-GFP probe [35] was diluted in blocking solution to 10 µg/mL, and samples were incubated at room temperature overnight. After five washes in PBS, IRDye 680RD-labeled goat anti-mouse antibody (#926–32,210, LI-COR Biosciences) was added in a 1:1,000 dilution and incubated for 2 h at room temperature. Coverslips were washed three times in PBS, sealed on a glass slide with a drop of antifade reagent (#9071S, Cell Signalling) and imaged as previously described. Four separate imaging fields were imaged and analyzed for each genotype, taking the mean Spearman correlation across all cells for each imaging field as a summary statistic for that field. and imaged as previously described. Foci quantification was performed in a blinded manner with ImageJ.

**Single-molecule fluorescence microscopy.** WT and Δ*ppk* Hfq-PAmCherry strains colonies were cultured in LB medium for 6 h at 37 °C and 200 rpm. Subsequently, the cells were diluted 1:400 into N+Gutnick and grown overnight under the same conditions. To induce nitrogen-starvation, the overnight culture was diluted in N-Gutnick to an OD_600_ ~ 0.02 and grown as above. About 5 h after inoculation, the cells entered N-starvation (N-0). Images were taken before (N+), at N-0 and at select timepoints after N-starvation. For imaging preparation, 0.5 ml of cells were centrifuged and resuspended in filtered spent N-Gutnick media. The cells were immobilized on 2% (w/v) agarose:spent N-Gutnick medium pads and imaged at room temperature on a wide-field Olympus IX71 inverted microscope equipped with a 100 × 1.40 NA oil immersion objective. Hfq-PAmCherry molecules were photoactivated with 50–100-ms pulses of 405-nm laser (Coherent Cube, 405−100; 2 W cm^-2^, followed by imaging under excitation by a 561-nm laser (Coherent Sapphire, 561−50; 0.34 kWcm^-2^). Frames were captured at 50 Hz using a 512 × 512-pixel Photometrics Evolve EMCCD camera.

**Single-molecule diffusion analyses.** The recorded Hfq-PAmCherry movies were subsequently analyzed using the SMALL-LABS algorithm [72] for single-molecule localization and tracking.

To determine the diffusion coefficient of individual trajectories, tracks of at least 7 frames analyzed and their mean-square-displacement to time lag τ = 5 were fit to the diffusion model MSD = 4*D*τ + 4*σ*^2^, where *D* is the associated apparent diffusion coefficient of the trajectory and *σ* is the localization uncertainty. Construction of representative PALM images was performed as previously described [73]. Briefly, Hfq-PAmCherry localizations with a 95% confidence interval less than 80 nm were used. A Gaussian kernel was applied to each localization, with the degree of blurring (*σ*) set to the confidence interval of that localization.

To determine the subpopulation of Hfq molecules associated with H-bodies, we classified Hfq-PAmCherry trajectories by their overlap with the H-body foci. To identify H-body foci, all frames for each movie were summed to generate a composite image; regions in this image where Hfq molecules are stably bound form distinct bright foci. The foci present in this image were detected by the Laplacian of Gaussian algorithm (*min_sigma* = 3, *max_sigma* = 5, *threshold* = 0.1), segmented, and assumed to be H-bodies. Putative foci were filtered to determine if they were indeed stable H-bodies: a focus was kept if it contained at least 10 trajectories of at least 10 frames each, and where at least 70% of the trajectory overlapped with the focus. The median trajectory length in our dataset is approximately 10 frames; therefore, this number was chosen for the minimum threshold of trajectory length in the filtering. To identify the different states of Hfq, single-molecule trajectories were then classified based on their overlap with foci areas. Trajectories were classified as ‘In’ if they completely overlapped with a focus area for their entire duration, ‘In/out’ if they overlapped with a focus for 25% to 99% of their duration, and ‘Out’ if they overlapped for less than 25% of their duration [74]. The squared displacement, *r*^*2*^, was calculated for consecutive localizations within a trajectory of at least 4 frames (*τ* = 20 ms). The cumulative probability of the squared displacements in the observation period *τ* (*P*(*r*^*2*^*, τ*)) was generated from the pool of squared displacements across multiple tracks for each sample replicate by counting the number of squared displacements less than or equal to *r*^*2*^ normalized by the sample size. The CDF of *r*^*2*^ was fit to analytical functions describing the diffusive processes with three dynamic states. *D*_*1*_, *D*_*2*_, and *D*_*3*_ are the diffusion coefficients for the different states, and *α* describes the relative fraction between the states.

$(\text{Three}-\text{state})\;P\left(r^{\text{2}},\;\tau \right)=\text{1}-\left(\alpha_{\text{1}}e^{\frac{-r^{\text{2}}}{\frac{\text{8}}{\text{3}}D_{\text{1}}\tau }}+\alpha_{\text{2}}e^{\frac{-r^{\text{2}}}{\frac{\text{8}}{\text{3}}D_{\text{2}}\tau }}+\left(\text{1}-\alpha_{\text{1}}-\alpha_{\text{2}}\right)e^{\frac{-r^{\text{2}}}{\frac{\text{8}}{\text{3}}D_{\text{3}}\tau }}\right)$

Here, we assumed the *D*_3_ population corresponds to freely diffuse molecules as *D*_3_ > 1 μm^2^/s, such that the fraction of freely diffuse molecules is $F_{\text{free}}=\alpha_{\text{3}}$. We assumed the two slower populations, *D*_1_ and *D*_2_, correspond to a combination of nucleoid-associated and within-condensate Hfq molecules. To identify the specific contributions from within-condensate molecules, we first analyzed the trajectories that were classified as “In”. The same diffusion analysis was performed as above but using a two-state model.


$$\begin{array}{c} (\text{Two}-\text{state})\;P\left(r^{\text{2}},\;\tau \right)=\text{1}-\left(\alpha e^{\frac{-r^{\text{2}}}{\frac{\text{8}}{\text{3}}D_{\text{1}}\tau }}+(\text{1}-\alpha )e^{\frac{-r^{\text{2}}}{\frac{\text{8}}{\text{3}}D_{\text{2}}\tau }}\right) \end{array}$$

The resulting *D*_1_ and *D*_2_ were consistent with the *D*_1_ and *D*_2_ from the entire trajectory dataset. Next we fit the combined “In” and “In/out” subsets to a three-state model and used the combined weight fractions *α*_1_ *+ α*_2_ as the within condensate contribution for this subset as *D*_3_ was consistent with freely diffuse molecules. The total fraction of Hfq in condensates is then defined as:


$$\begin{array}{c} F_{\text{condensate}}=\frac{N_{\prime \text{In}\prime \;}+\;N_{\prime \text{In}/\text{out}\prime \;}}{N_{\text{total steps}}}\cdot \left(\alpha_{\text{1}}+\alpha_{\text{2}}\right) \end{array}$$

Since it is possible for a condensate focus to overlap with the nucleoid, we cannot exclude that some amount of *F*_condensates_ is also nucleoid-associated. We defined the fraction of Hfq that is only nucleoid-associated and not within a condensate as:


$$\begin{array}{c} F_{\text{nucleoid}}=\text{1}-\;F_{\text{free}}-\;F_{\text{condensate}}\; \end{array}$$

We note that the reported population fractions are percentages of the total Hfq displacements analyzed.

**PolyP extraction and detection.** PolyP was extracted as previously described [75] with modifications. Briefly, WT and *Δppk* bacteria cultured overnight in N+ medium was reinoculated into N− medium and grown at 37 °C. At exponential phase (OD_600_ = 0.5), N-0, N-3, N-6, N-12, N-18 and N-24, cultures equivalent to 4 mL OD_600_ = 1.0 were collected, spun down, washed in PBS and resuspended in 350 µL AE buffer (50 mM sodium acetate, 10 mM EDTA). Suspension was sonicated on ice, and 300 µL phenol and 40 µL 10% SDS was added. The mixture was incubated at 65 °C for 5 min, and then on ice for 2 min. 300 µL chloroform was then added, and the mixture was spun down. The upper aqueous phase was mixed with 350 µL chloroform, spun down, and aqueous phase was again collected. After measuring sample volume, 3 times volume of 100% ethanol, 10% volume of 3 M sodium acetate and 5 µL 5 mg/mL glycogen (#AM9510, Thermo Fisher) was added. The mixture was vortexed and incubated on ice for 1 h. DNA, polyP and glycogen was pelleted, washed with 70% ethanol and air dried. Dried samples were dissolved in 500 µL MP buffer (125 mM NaCl, 5 mM MgCl_2_, 25 mM Tris-HCl pH 7.5). Per 50 µl sample, 1 µl M-SAN nuclease (#70950-202, ArcticZymes) was added and incubated at 37 °C for 2 h. To examine polyP on gel, 10 µL sample was mixed with 2 µL 20% glycerol and 2 µL gel loading dye, loaded onto a 4%–20% TBE gel (#EC6225Box, Invitrogen), and ran for 4 h at 100 V. PolyP 300mer and 130mer were loaded as chain length markers. To measure polyP concentration (in phosphate units), 30 µL M-SAN digested samples were added with 1 µl *Saccharomyces cerevisiae* exopolyphosphatase (yPpx) and incubated at 37 °C for 2 h. Potassium phosphate solutions ranging from 0 to 250 µM was used as standards. In a microplate, 25 µL of each standard was mixed with 25 µl MP buffer, 15 µl 5× yPpx buffer (100 mM Tris-HCl pH 7.5, 25 mM MgCl_2_, 250 mM ammonium acetate), 10 µL water and yPpx. Twenty-five µL of each sample was mixed with 50 µL water. Molybdate working solution was prepared by mixing 91.2% total volume of detection base (0.6 mM antimony potassium tartrate, 600 mM H_2_SO_4_, 2.4 mM ammonium heptamolybdate) and 8.8% total volume of 1 M ascorbic acid. Twenty-five µL working solution was added to each standard and sample well, rested for 2–5 min, and absorbance at 882 nm was measured.

**ATP measurements.** About ~1 OD mL culture of MG1655 WT or Δ*ppk* was collected at N+ or N-24, boiled at 95 °C for 20 min and spun down. The CellTiter-Glo 2.0 Assay kit (#G9241, Promega) was used to determine ATP concentration in the supernatant. Briefly, supernatant was mixed with pre-equilibrated CellTiter-Glo 2.0 Reagent at a 1:1 ratio in an opaque walled 96 well plate. Plate was incubated at room temperature for 10 mins to allow the signal to stabilize after which luminescence was measured on an Infinite M1000 (Tecan). To extrapolate ATP concentrations, a standard curve was generated using purified ATP (#R0441, Thermo Scientific) in the same media that the cells were cultured in. ATP concentrations were normalized to protein concentrations in the pellets.

**Immunoblotting.** Strains of interest were grown to various stages of N-starvation, and cultures equivalent to 1 ml OD_600_ = 0.6 were collected, spun down, washed and resuspended in 50 µL SDS-polyacrylamide gel loading buffer, and lysed by incubating at 95 °C for 10 min. Protein samples were then run on a 4%–12% NuPAGE Bis-Tris gel (#NP0336BOX, Thermo Fisher) at 175 V for 45 min. The gel was then transferred onto a polyvinylidene difluoride membrane (#1620174, Bio-Rad) and blocked with 5% milk in TBST for ≥1 h. The membrane was incubated in a dilution of 1:1,000 anti-mCherry monoclonal antibody (#M11217, Thermo Fisher) or 1:1,000 anti-GFP polyclonal antibody (#632592, Takara Bio) in blocking solution for ≥1 h, rinsed five times with TBST, and then incubated in 1:10,000 diluted anti-rat or anti-rabbit secondary antibodies labeled with IRDye 680RD or 800CW (#926-68076 and #925-32211, LI-COR Biosciences). The membrane was rinsed three times in TBST, once in TBS, and imaged on a LI-COR Odyssey CLx imager (LI-COR Biosciences). All washes and incubations were done at room temperature. A gel was run in parallel and stained with Coomassie blue as normalization control. Image quantification was performed using ImageJ, and three biological replicates were included in each experiment.

**Native western blot.** Bacterial cultures equivalent to 40 ml OD_600_ = 1.0 were collected, spun down, and lysed by sonication in 5 mL native lysis buffer (20 mM Tris-HCl pH 8, 1 mM β-mercaptoethanol, 1 mM EDTA, 2% glycerol and cOmplete protease inhibitor (#5056489001, Sigma-Aldrich)). Samples were centrifuged to remove insoluble debris, and supernatant was mock treated or digested with DNase I (#18068015, Invitrogen), RNase A (#10109169001, Sigma-Aldrich) or yPpx for 2 h at 37 °C before running on a 4%–20% TBE gel (#EC6225Box, Invitrogen) at 100 V for 16 h. The gel was incubated in SDS-PAGE running buffer for 20 min and was subjected to western blotting following standard protocols described above.

**Fractionation of soluble and insoluble proteins.** As previously described [76], cultures equivalent to 4 ml OD_600_ = 1.0 were collected by centrifugation. The pellet was resuspended in 50 µL ice-cold lysis buffer (10 mM potassium phosphate pH = 6.5, 1 mM EDTA, 20% sucrose, 1 mg/mL lysozyme, 50 µ/ml benzonase), incubated for 30 min on ice, and frozen at −80 °C. After thawing on ice, 360 µL ice-cold buffer A (10 mM potassium phosphate pH = 6.5, 1 mM EDTA) was added. The mixed sample was transferred to a 2 mL tube containing 200 µL 0.5 mm glass beads and was incubated for 30 min at 8 °C with 1,400 rpm shaking. After settling down the beads, 200 µl lysate was centrifuged at 16,000*g* for 20 min, and supernatant (soluble proteins) was collected for further use. The pellet (insoluble proteins) was washed with buffer A, buffer B (buffer A containing 2% Nonidet P-40), and again with buffer A. Previously collected supernatant was mixed with ¼ volume of 100% trichloroacetic acid, incubated for 10 min at 4 °C and spun down at 21,000*g*, and precipitates were washed three times with ice-cold acetone. After washing acetone was removed from samples by heating at 37 °C. Both soluble and insoluble proteins were resuspended in 100 µL 1× reducing SDS buffer (6.5 mM Tris-HCl pH = 7.0, 10% glycerol, 2% SDS, 0.05% bromophenol blue and 2.5% β-mercaptoethanol), boiled at 95 °C for 10 min, and wash subjected to further analysis as described above.

**Protein purification.** As described previously [22], *E. coli* BL21 (DE3) carrying pET-21a-*hfq*, or pET-21a-*hfqS65C* were grown in LB + ampicillin at 37 °C to OD_600_ = 0.5. Protein expression was induced with 0.1 mM isopropyl-β-D-thiogalactopyranoside (IPTG). After 16 h at 22 °C, cells were pelleted (5,000*g*, 20 min, 4 °C) and resuspended in lysis buffer (25 mM Tris, 300 mM NaCl, DNase I (5 mg/ml), 5 µL of Benzonase nuclease (Merck), 100 µM MgCl_2_, and cOmplete (Roche) (pH 7.5)) and lysed by a 3 min sonication at 4 °C. Lysates were incubated at room temperature for 30 min, boiled in water for 20 min to precipitate most bacterial proteins, and transferred to room temperature water bath for 15 min. Lysate was spun at 30,000*g*, 30 min, 4 °C, and supernatant was loaded onto two connected 5 mL HisTrap columns (Sigma-Aldrich). The columns were washed with 20 mL of buffer A (300 mM NaCl, 25 mM Tris pH 7.5), 60 mL of 6 M guanidinium hydrochloride in buffer A, a 120 mL gradient from 6 to 0 M guanidinium hydrochloride in buffer A, followed by 60 mL of buffer A. Hfq was eluted with a 70 mL gradient from 15 to 300 mM imidazole in buffer A. The fractions containing Hfq were pooled and incubated for 1 h with 100 µM MgCl_2_ and 4 µL of Benzonase before overnight dialysis against buffer A. After dilution the sample 1:1 into 25 mM Tris pH 7.5, the sample was loaded onto a 5 mL HiTrap SP HP column (Sigma-Aldrich) and washed with 5% buffer B (100 mM NaCl, 25 mM Tris pH 7.5). Hfq was eluted with a gradient from 6% to 40% buffer C (1 M NaCl, 25 mM Tris pH 7.5). Purified samples were pooled, dialyzed against buffer A, and stored at −80 °C.

**PolyP and Hfq labeling.** PolyP-300 was provided by T. Shiba (RegeneTiss, Japan). All chemicals were from Sigma-Aldrich unless stated otherwise. PolyP-300 was labeled with AF647 as previously described [22]. Briefly, 37.5 mM polyP-300 was incubated with 1 mg AF647 cadaverine (Life Technologies) and 200 mM 1-ethyl-3-(3-dimethylaminopropyl) carbodiimide (Invitrogen) in 100 mM NaCl and 20 mM MOPS pH 8.0 at 45 °C for 1 h before quenching on ice. Free dye was removed using a PD-10 column (GE Healthcare) equilibrated with the same buffer. Flow through was cleaned up using Zeba desalting column (Qiagen). PolyP concentration was determined in 100 µL samples containing the same buffer and 1 µg of DAPI, using excitation and emission wavelengths of 415 nm and 550 nm, respectively. Unlabeled polyP-300 was used as standard. Labeling efficiency (0.1%–0.2%) was quantified with AF647 fluorescence in the same samples. HfqS65C was labeled with Cy3 Maleimide Mono-Reactive Dye (Amersham) following the manufacturer’s protocol. Briefly, ~1 mg of purified HfqS65C was reduced with 180 µg Tris-(2-carboxyethyl)phosphine hydrochloride (TCEP) in 100 mM NaCl and 50 mM K_2_HPO_4_ pH 7.5. Dye was dissolved in 50 µL of anhydrous dimethyl sulfoxide and added to reduced HfqS65C yielding a final reaction volume of 1 mL. The reaction was incubated for 2 h at room temperature and then overnight at 4 °C. Excess dye was removed by a PD-10 desalting column packed with Sephadex G-25 resin equilibrated with the same buffer. The labeled protein was eluted and concentrated using an Amicon Ultra centrifugal filter with 3-kDa MW cutoff. Protein concentration and labeling efficiency (~30%) were calculated using a molar extinction coefficient of 150,000 M^−1^cm^−1^ at 552 nm for Cy3 and a correction factor of 0.08 for dye absorbance at 280 nm.

**Native gel electrophoresis and electrophoretic mobility shift assay.** Twenty-five µM Hfq was incubated with varying concentrations of FAM-rA_30_ and/or AF647-polyP-300 in 200 mM NaCl and 25 mM Tris pH 7.5 at room temperature. Samples were mixed with glycerol and loading dye and run on a tris-borate EDTA (TBE) 4% to 20% gel (Invitrogen) at 100 V for 4 h on ice using TBE buffer. RNA and polyP were visualized using a fluorescence gel imager. Hfq was visualized using Coomassie blue stain.

**In vitro condensate reconstitution.** Purified Hfq (supplemented with 4% Cy3-Hfq S65C) was prepared in 50 mM NaCl and 20 mM HEPES pH 7.0 in the presence or absence of FAM-rA_30_ and/or AF647-polyP-300. To generate the phase diagram, purified Hfq was prepared in 50 mM NaCl and 20 mM HEPES pH 7.0 in the presence or absence of the indicated concentrations of polyP-300. Concentrations of all biomolecules were indicated in figure legends. Fifty µL sample was mixed and imaged in 16-well CultureWell slides (#112358, Grace Bio-Labs) on the SP8 confocal microscope. Slides were treated with 5% (w/v) pluronic acid (Thermo Fisher) overnight and washed with ddH_2_O prior to sample preparation. Quantitative analysis of the phase diagram was conducted using an automated pipeline. The raw images were normalized using the equalize_adapthist function of the python skimage module with CLAHE limits imposed. The normalized image was then blurred with a Gaussian filter, and edges were detected using the canny method of the skimage feature module. A disk with a radius of 3 pixels was used to close features, and then all enclosed regions with an area of at least 150 px were considered to be candidate condensates. We further imposed a series of circularity constraints on each candidate condensate to filter potential noise: the circularity (ratio of 4 * Π * area/perimeter^2) was required to be at least 0.4, the ratio of major to minor axis required to be less than 1.4, the eccentricity required to be less than 0.8, the solidity required to be greater than 0.9, and the extent to be between 0.55 and 0.95 (using skimage-calculated features). We collected both the number and the total area of the condensates in each field of view (with 3–5 imaging fields collected for each condition). We then applied the Otsu thresholding method to the distributions of log-scaled condensate counts and total condensate areas (at the level of ALL imaging fields) to identify thresholds for what constituted ‘enough’ condensates (according to either of those metrics) to indicate phase separation. For each condition, we then took the median of the condensate counts and areas across the imaging fields and indicated condensates to be present if either (or both) of the count or total area thresholds were exceeded.

**Fluorescence in situ hybridization.** As described previously [16], N-24 WT and Δ*ppk hfq::hfq-mCherry* strains were directly fixed in culture by 4% formaldehyde (#18814, Polysciences) at room temperature for 15 min and on ice for 30 min. Cells were then washed three times with chilled PBS, and resuspended in GTE buffer (50 mM glucose, 10 mM EDTA pH 8.0, 20 mM Tris-HCl pH 7.5) containing lysozyme and RNase inhibitor. Samples were loaded on poly-L-lysine treated coverslips and briefly incubated, washed three times with chilled PBS, once with chilled 80% methanol and once with chilled acetone. After drying for 5 min at 37 °C, coverslips were washed once with chilled 50% ethanol and dried again at 37 °C. Samples were then washed twice with 2×SSCT (0.3 M NaCl, 30 mM Na citrate, 0.1% Triton X-100), incubated in 2×SSCT + 50% formamide at 37 °C for 30 min. For each sample, 2 µL of AF647-labeled probe (IDT) was added to 20 µL hybridization buffer (3×SSC, 50% formamide, 10% dextran sulfate and RNase inhibitor) and loaded on coverslips. Samples were incubated at 94 °C for 2 min and then at 55 °C overnight under hydrated conditions. Coverslips were washed twice with 2×SSCT + 50% formamide for 30 min at 37 °C, once with 2×SSCT + 25% formamide for 10 min at room temperature, three times with 2×SSCT for 10 min, and once with PBS for 10 min. Coverslips were treated again with 4% formaldehyde and DAPI for 30 min at room temperature. They were then washed with PBS, 80% methanol and three times again with PBS at room temperature. Coverslips were sealed and imaged as described in previous sections.

### Rifampicin treatment, sequencing, and data analysis for RNA stability measurements

*Growth and harvest*—N-24 WT and Δ*ppk E. coli* were supplemented with 150 µg/mL rifampicin (#R3501, Sigma-Aldrich) and incubated at 37 °C with 200 rpm shaking. Samples were collected before rifampicin addition, or at 10, 30 and 60 min after treatment, and were immediately mixed with RNAProtect Bacteria Reagent (#1018380, Qiagen); for the post-treatment timepoints, a separate untreated control was collected after the same lag time. Cells were pelleted and stored at −80 °C. Each sample was also plated for CFUs at the time of harvest in order to provide a normalization factor (used below). Three biological replicates were performed for each genotype/condition combination.

*Lysis and RNA purification*—On ice, each sample pellet was resuspended in 90 µl of 1× TE (10 mM Tris pH 7.0, 1 mM EDTA pH 8.0), briefly centrifuged to remove liquid from tube walls and transferred to 1.5 ml tube and then transferred to 1.5 ml microfuge tubes. Then 1 µl of Ready-Lyse Lysozyme Solution (LGC Biosearch Technologies) was added to each sample and mixed. Samples were incubated at 30 °C for 15 min and returned to ice. Then, 10 µl of Proteinase K (Thermo-Fisher) was added to each sample and mixed. Samples were incubated at 23 °C for 15 min, with resuspension every two minutes then returned to ice. ERCC RNA spike-in control mix (Ambion) was diluted 1:25 in water as one batch (8.6 µl of ERCC, 206.4 µl water). Then, 5 µl of the diluted mix was added to each sample, except for *Δppk t* = 60 (no rifampicin) replicate 1, which received 1.6 µl instead of 5 µl. After vortexing the samples, they were clarified by centrifugation (12,000 r.c.f., 30 s) and up to 250 µl of clarified lysate was transferred to a new tube. The samples were cleaned using RNA Clean & Concentrator-96 (Zymo Research) per manufacturer’s directions for total RNA clean-up and eluted with 25 µl of water per sample. Each sample was transferred to tubes that contained 60 µl of water, 10 µl of 10× Baseline Zero DNase buffer, 5 µl of Baseline Zero DNase (LGC Biosearch Technologies), and 2 µl of RNase Inhibitor, Murine (recombinant, NEB) in each tube. Samples were incubated at 37 °C for 30 min. The samples were re-cleaned using RNA Clean & Concentrator-96 (Zymo Research) per manufacturer’s directions for total RNA clean-up and eluted with 20 µl water per sample. The concentration of RNA was determined using 5 µl samples and Quantifluor RNA system (Promega) per manufacturer’s directions for quantitating RNA in multiwell plates.

*Library preparation and sequencing*—Appropriate amounts of nuclease-free water were added to 0.1 µg of RNA per sample to achieve 11 µl of starting material for NEBNext rRNA Depletion Kit (Bacterial, NEB). The kit was used per manufacturer’s directions to remove rRNA from the samples. Library preparation was performed using NEBNext Ultra II Directional RNA Library Prep Kit for Illumina (NEB). Samples were prepared per kit directions except that 2.5 µl of diluted (1:100) NEBNext Unique Dual Index UMI Adaptors DNA Set 1 was used instead of the NEBNext Adaptor for Illumina, and that Omega Mag-Bind TotalPure NGS Beads were used instead of NEBNext Sample beads for post second-strand synthesis, post adapter ligation, and post PCR clean-up steps. Pooled libraries were subjected to sequencing on a NextSeq 2000 instrument.

*Data analysis*—After initial read preprocessing, alignment and QC using the IPOD-HR processing pipeline (equivalent to that described above for the RNA-seq samples), overlaps of aligned reads to genes were quantified using the summarizeOverlaps() function of the R package GenomicAlignments (version 1.26.0) running under R 4.0.2, with options “singleEnd = FALSE, inter.feature = FALSE, ignore.strand = TRUE, mode = ’IntersectionStrict’,fragments = TRUE”. A composite reference transcriptome including both all annotated MG1655 transcripts and the transcripts from the ERCC spike-in set was used. Read counts for each feature (gen) were then normalized by the total read count for that sample, and then sequencing depths of genomic features further rescaled by the median abundance of ERCC transcripts in that sample (considering only ERCC transcripts with a relative abundance greater than 5*10^−4^) in order to provide a relative estimate of per-cell transcript abundance. For each gene, we then fitted an exponential decay equation (*y* = a*exp(−1*t/*b*), where *y* is the normalized transcript abundance, *a* and *b* are coefficients to be fitted, and *t* is the time) to the data for each time course, using the scipy.optimize.curve_fit function from scipy 1.8.0, using the ‘dogbox’ optimizer with both parameters constrained to be non-negative, and *a* and *b* initialized to 0.001 and 20, respectively; *a* represents a baseline abundance (at *t* = 0) for each transcript, and *b* is the exponential decay time constant (in minutes); we then considered for further analysis only those transcripts for which the q-value of the main decay constant was less than 0.1 (indicating a reasonably confident fit). We considered ‘measurable’ decay constants to be those between 5 min and 65 min.

It is important to note that the half-life distributions observed in our experiments are substantially longer than those that have been previously measured for late exponential/early stationary phase *E. coli* and *Salmonella* (e.g., [77,78]). There are both biological and methodological reasons likely contributing to these differences. On the biological side, we are targeting an unusual stress condition (long term nitrogen starvation), which has previously not been heavily investigated, and in which cells must surely have become dormant to a much greater extent than the growth conditions typically used in prior transcription shutdown experiments (and in particular, the cells used in classic experiments were often under periods of rapid physiological change during which rapid transcriptome turnover could well be a benefit to the cells). From a technical standpoint, we deliberately chose to take a more extended series of timepoints (15 min increments instead of 2 min increments) in part because were studying a long-term stress response in truly non-dividing cells; this process may bias us toward confident measurements of long-lived transcript stabilities versus short-lived transcript stabilities, or might cause us to miss short-timescale decay of transcripts and instead focus on longer term behavior. Nevertheless, we were able to obtain confidently fitted models (R^2^ > 0.5 and *q* < 0.1) for the transcripts corresponding to more than half of the genes in *E. coli* (2,743) in the RIF-treated conditions, arguing for our ability to make confident and reliable measurements of the decay processes at work in cells under long term nitrogen starvation, and in particular the long-term stability of transcripts under those conditions.

**Untemplated poly(A) tail length determination.** WT and Δ*ppk* Hfq-mCherry strains were streaked on LB agar plates from cryogenic storage and grown at 37 °C. Colonies (four biological replicates per strain) were inoculated into N+ Gutnick medium and grown overnight at 37 °C with shaking at 200 rpm. Overnight culture was back-diluted to OD600 = 0.01 in 250 mL N- Gutnick medium. At OD600 = 0.4 ~ 0.45 (N+) and 24 h after nitrogen-starvation (N-24), cultures equivalent to 40 ml OD600 = 1.0 were collected and immediately spun down for 3 min at 13,000*g* at 4 °C in a fixed-angle rotor. Cell pellets were shock-frozen in a dry ice-ethanol bath, and stored at −80 °C.

*Sequencing Library Preparation.* RNA extraction and cleaning: To lyse cells, 700 µl of DNA/RNA Shield (Zymo Research, R1100-50) was added to each pellet, cells were resuspended, incubated at room temperature for 5 min, and then frozen solid at −80 °C. Upon thawing, lysate was transferred to 2 mL tubes and clarified via centrifugation (12,000 r.c.f., 5 min). The soluble fraction was transferred to new tubes. Samples were cleaned using RNA Clean & Concentrator-96 (Zymo Research) per manufacturer’s directions for RNA clean-up from samples in DNA/RNA Shield. The cleaned samples were digested with BaselineZero DNase (1× Baseline Zero buffer, 10 µL of Baseline Zero enzyme, 2 µL of Murine RNase Inhibitor (NEB, M0314) for 30 min at 37 °C. Samples were cleaned using an RNA Clean & Concentrator-96 (Zymo Research) per manufacturer’s directions for total RNA clean-up. The cleaned samples were digested a second time with BaselineZero DNase (1× Baseline Zero buffer, 10 µL Baseline Zero enzyme, 2 µL of Murine RNase Inhibitor (NEB, M0314)) for 60 min at 37 °C. RNA was cleaned by the addition of 1.8× volumes of RNAClean XP beads (Beckman Coulter), mixing, incubating on ice for 15 min, removing the unbound fraction, washing undisturbed beads with 200 µL of 80% ethanol twice, removing the ethanol and allowing evaporation of the residual liquid, followed by elution of RNA in 25 µL of water. Samples were stored at −80 °C.

RNA ligation and conversion to dsDNA: For each sample, 1 µg of RNA and water to reach a 40 µL total volume were added to a PCR strip tube. The 5′ phosphates were removed from the RNA by rSAP (1× rCutsmart buffer, 3 units of rSAP, (NEB, M0371)); 80 units of murine RNase Inhibitor (NEB, M0314) incubated for 2 h at 37 °C. RNA was cleaned by the addition of 1.8× volumes of RNAClean XP beads (Beckman Coulter), mixing, incubating on ice for 15 min, removing the unbound fraction, washing undisturbed beads with 200 µL of 80% ethanol twice, removing the ethanol and allowing evaporation of the residual liquid, followed by elution of RNA in 7 µL of water. The RNA was transferred to new PCR strip tubes. T4 RNA ligase I (30 units, NEB, M0437M) was used to ligate a 5′ phosphorylated, 3′-dideoxy terminated, UMI containing adaptor oligo (100 pmol of /5Phos/TCAAGCAGTAGNNNNNNNNCAGCAGT

TCGATAAGCGG/3ddC/, synthesized by IDT) to the 3′ ends of the RNA in a buffered reaction (1× T4 RNA Ligase Reaction Buffer, 12.5% PEG 8,000, 1 mM ATP, 40 units of murine RNase Inhibitor, 1/10 volume DMSO) at 16 °C for 12–14 h. The ligation product was cleaned by the addition of 1.8× volumes of RNAClean XP beads, mixing, incubating on ice for 15 min, removing the unbound fraction, washing undisturbed beads with 200 µL of 80% ethanol twice, removing the ethanol and allowing evaporation of the residual liquid, followed by elution of RNA-DNA in 7 µL of water. The RNA-DNA eluate was then transferred to new PCR strip tubes. The RNA of the RNA-DNA was fragmented in buffer (4 µL of 5× Protoscript II buffer, M0368S, NEB; 1 µL of 10 mM dNTPs) with a primer complementary to the adaptor oligo (5’CCGCTTATCGAACTGCTG, 20 pmol per reaction) at 94 °C for 6 min prior to reverse transcription. The RNA-DNA was reverse transcribed using ProtoScript II Reverse Transcriptase (200 units of M0368S, NEB) which was mixed into each reaction after the addition of DTT (10 mM final) and murine RNase Inhibitor (8 units) to each sample. The reactions were incubated 25 °C for 5 min, 42 °C for 1 h, 80 °C for 15 min, and then 4 °C. After samples were placed on ice, 8 µL of NEBNext Second Strand Synthesis Reaction Buffer with dUTP (E7426AA, NEB), 1 µL of random primers (E7422AA, NEB), and 47 µL of water were mixed well into each 20 µL RT reaction. Then 4 µL of NEBNext Second Strand Synthesis Enzyme (E7425AA, NEB) was mixed into each reaction. Reactions were incubated at 16 °C for 1 h. The dsDNA was cleaned by the addition of 1.8× volumes of Mag-Bind TotalPure NGS beads (Omega Biotek), mixing, incubating 5 min RT, removing the unbound fraction, washing undisturbed beads with 200 µL of 80% ethanol twice, removing the ethanol and allowing evaporation of the residual liquid, followed by elution of the DNA in 50 µL of water.

Library preparation and sequencing: The DNA was prepared for sequencing using NEBNext Ultra II DNA Library Prep Kit for Illumina (E7645L, NEB) with NEBNext Multiplex Oligos for Illumina Dual Index Primers Set 1 (E7600S, NEB) per manufacturer’s protocol (with a 1–10 dilution of the standard adapter) except the blue capped USER enzyme (E7428AA, NEB) was used instead of red capped USER enzyme (E7602AVIAL, NEB), Mag-Bind TotalPure NGS beads (Omega Biotek) were used instead of NEBNext Sample Purification Beads, and 14 cycles of amplification were performed at the PCR stage. Pooled reactions were sequenced using NextSeq 1000 Sequencing System (Illumina) using a P2 100 cycle kit with 75 bp read 1, 25 bp read 2, 19 bp index 1, and 8 bp index 2 reads.

*Data analysis.* Reads were initially pre-processed and aligned using the same procedures as those noted above for the standard RNA-seq datasets, with the crucial exception that reads were aligned in local, rather than end-to-end, mode. Apart from this, the number of ‘T’ bases in the read (which, due to the location/orientation of our sequencing adapter, correspond to 3′-end ‘A’ bases in the original RNA) was taken to be the number of terminal As on that read. For each gene, we then aggregated the distribution of 3′-terminal ‘A’ counts occurring on reads mapping (in the correct orientation) to any position from 50 bp before to 250 bp after the end of that gene, thus assigning reads occurring in that 3′ window to the gene that they are close to the end of. We note that while we could instead have performed this analysis at the level of annotated transcripts/transcriptional units, it appeared likely to us that in many cases we might miss polyA tail assignments due to imperfect knowledge/enumeration of all transcripts produced under the conditions being studied, whereas the primary weakness with our current approach is that we might assign to a single gene a polyA state that also affects other genes in the same operon.

For each gene, we then fitted zero-inflated negative binomial regression models to the distributions of aggregated polyA tail length observations across the 16 experiments performed here (four biological replicates for each of four conditions). We incorporated coefficients contributing to the mean polyA tail length for the possible combinations of *ppk* genotype and nitrogen-starvation status (four degrees of freedom) and fitted only a single zero inflation rate and overdispersion parameter for each gene. Fits were performed using the python statsmodels package, version 0.13.2, with default parameters except that we allowed a maximum of 500 iterations for coefficient fitting.

**GO term enrichment analysis.** All GO term enrichment analysis was performed using the iPAGE 1.2b package [79] using default settings except as otherwise noted. The GO term annotation set used was derived from the Uniprot [80] MG1655 reference proteome indexed by b-number for any nucleic acid based analysis, and for all Uniprot proteins associated with *E. coli* K12 (taxon ID 83333) indexed by Uniprot identifier for the mass spectrometry analysis. For continuous inputs (e.g., log2 fold changes), inputs were split into equally populated bins for the mutual information analysis; for discrete inputs, bins were allocated as shown.

**Hfq-mCherry immunoprecipitation.** Modified protocol of the RFP-Trap Magnetic Agarose beads (#rtma, Proteintech) was used to perform anti-mCherry immunoprecipitation. Briefly, *E. coli* MG1655 *hfq::hfq-mCherry*, WT or *Δppk*, were grown in N- Gutnick medium to log phase or N-24. An mCherry-only control was used to assess non-specific binding. 10–15 ODmL of bacteria was collected by centrifugation for every replicate. The bacteria were lysed in RIPA buffer (10 mM Tris-HCl pH 7.5, 150 mM NaCl, 0.5 mM EDTA, 0.1% SDS, 1% Triton X-100, 1% deoxycholate) with DNAse A and DNAse A buffer (#E1011, Zymo Research,). 600 μL buffer was used for 10 OD of bacterial pellet. Bacteria were lysed in Omni International Bead Beater Elite bead mill homogenizer using 0.1 mm zirconia beads. Beads were settled by centrifugation at 3,000*g* for 1 min, and the supernatant was removed in a fresh tube. The lysate was centrifuged at 16,000 *g* for 15 min at 4 °C to remove debris. Supernatant was saved, and BCA assay was performed for protein estimation and input normalization. RFP Trap magnetic agarose beads were prepared by washing 50 μl of beads in 1 mL Dilution buffer (10 mM Tris-HCl pH 7.5, 150 mM NaCl, 0.5 mM EDTA). Buffer was washed away using magnetic separation, followed by the addition of 400 μL of lysate and 600 μL of dilution buffer. Beads were incubated overnight at 4 in a nutator. The next day, beads were washed twice in the wash buffer (10 mM Tris-HCl pH 7.5, 150 mM NaCl, 0.05% Nonidet P40, 0.5 mM EDTA) by using magnetic separation followed by a wash in the Wash buffer without NP40. Twenty% of the beads were aliquoted in different tube, eluted in 30 μL 2× SDS buffer (120 mM Tris-HCl pH 6.8, 20% glycerol, 4% SDS, 0.04% bromophenol blue, 10% β-mercaptoethanol). These samples were run on a Biorad 4%–20% stain-free TGX gel. Stain-free images were collected after the gel run, and the gel was further processed for anti-mCherry western blotting. The rest of the beads (80%) were separated using magnetic separation and froze at −80 °C before sending them for Mass spectrometry.

**Gel excision and mass spectrometric (MS) analysis.** For MS analysis of native gel, samples were collected and lysed in the same manner as native western blot. Two 4%–20% TBE gels (#EC6225Box, Invitrogen) were run in parallel at 100 V for 1.5 h. One gel was subjected to native western blotting to identify the location of Hfq-mCherry oligomers, while the other gel is stained in warmed Coomassie blue for 5 min, destained overnight, and washed with ddH_2_O for 24 h. Area corresponding to the Hfq-mCherry oligomers was cut out from the WT N-24 lane, while areas of the same migration distance on other lanes were also collected. For analysis of co-IP samples, samples were run on a 4%–12% NuPAGE Bis-Tris gel (#NP0336BOX, Thermo Fisher) at 175 V for 12 min, and stained as above. Gels were diced and washed repeatedly in 25 mM triethylammonium bicarbonate (TEAB, #T7408, Sigma-Aldrich) in 50 v/v % acetonitrile (ACN, #34998, Sigma-Aldrich) followed by 100% ACN. Gel pieces were then incubated in 25 mM TEAB and 10 mM dithiothreitol (#D11000, Research Products International) at 57 °C for 1 h, followed by iodoacetamide (#I6125, Sigma-Aldrich) at room temperature for 45 min. Gel pieces were dehydrated stepwise with 25 mM TEAB, 25 mM TEAB in 50% ACN and 100% ACN. After overnight digestion at 37 °C with trypsin (#V5111, Promega), reaction was quenched with formic acid, and supernatant was collected. The remaining gel pieces were washed with 25 mM TEAB followed by 100% ACN, with both supernatants collected and pooled together. Pooled supernatants were dried in speed vacuum with heating and dissolved in 100 mM TEAB supplemented with formaldehyde and sodium cyanoborohydride. For dimethyl labeling, different samples were either labeled with light (CH_2_O, #252549, Sigma-Aldrich) or medium (CD_2_O, #sc-228228, Santa Cruz Biotechnology) formaldehyde. Corresponding light- and medium-labeled samples were subjected to electrospray ionization mass spectrometry on an Orbitrap Fusion Lumos Mass Spectrometer (Thermo Fisher). Results were obtained by searching against the *E. coli* strain K12 peptide database (S7 Data).

**GO term comparison with P-bodies/stress granules.** We identified human proteins that are components of P-bodies and/or SGs based on “Gold standard” entries in the RNA granule database (https://rnagranuledb.lunenfeld.ca/). GO terms were assigned to the (*E. coli*) H-bodies, (human) P-body, and (human) SG proteins based on annotations from UniProt. We then used custom-written python code to identify the sets of GO terms present at the two-way and three-way interfaces of the GO term lists. We assessed significance via a permutation test in which we simulated the null distribution by randomly reassigning the set of *E. coli* K12 proteins assigned to Hfq/polyP foci and calculating the GO term overlaps with P-bodies and/or SGs for each of 1,000 permutations.

**Mammalian cell culture, plasmids and transfection.** HEK293 cells (ATCC CRL-1573, ATTC, Manassas, VA, USA), HeLa cells (ATCC CCL-2, ATTC, Manassas, VA, USA) and NIH3T3 cells (ATCC CRL-1648, ATTC, Manassas, VA, USA) were grown and maintained in DMEM (Gibco 11995065) supplemented with 10% w/v Fetal Bovine Serum (#F4135, Sigma-Aldrich) and 1% w/v Penicillin-Streptomycin (#SV30010, Cytiva*)*. All cells were cultured in a 37 °C incubator at 5% CO_2_. EcPPK1 was a gift from Michael Downey (Addgene plasmid # 108850). Transfections were carried out on cells seeded on glass coverslips in a 24-well plate using Lipofectamine LTX Reagent with PLUS Reagent (#15338100, ThermoFisher Scientific) as per the protocol provided on the manufacturer’s website. Cells were transfected for 24 h prior to treatment with sodium arsenite.

**Generation of yPpx expressing stable cell lines.** yPpx fused to a 3×FLAG tag on the N-terminus was cloned into the pLVX-pTuner Green vector (# 632176, Takara Bio). The vector contained the coding sequence for an additional N-terminal destabilization domain (DD), which causes the degradation of the protein in the absence of the small molecule Shield1 (#632189, Takara Bio). Lentiviruses were generated with this construct at The Vector Core (University of Michigan Medical School). NIH3T3 cells were transfected with the DD-3×-FLAG-yPpx lentivirus in the presence of polybrene (#TR-1003-G, EMD Millipore) at a concentration of 10 µg/ml. Cells were transfected for 24 h and allowed to recover for 24 h after removal of transfection media. Positively transfected clones were sorted based on ZsGreen fluorescence with the Cytoflex SRT (Beckman Coulter) using the B525 channel. Sorted cells were grown in the presence or absence of 0.5µM Shield1. The expression of yPpx was verified by western Blotting using a mouse anti-FLAG M2 antibody (#F3165, Sigma-Aldrich).

**Mammalian cell immunofluorescence staining and microscopy.** To visualize P-bodies and SGs via fluorescence microscopy, transfected cells were detached using 0.25% w/v Trypsin-EDTA (#T4049, Sigma-Aldrich) and seeded onto 12 mm circular coverslips (#72230-01, Electron Microscopy Sciences) placed in a 24-well plate at a density of 50,000 cells/well. The cells were treated with buffer or 0.5 mM sodium arsenite (#S7400, Sigma-Aldrich) for 30 min. Afterwards, the cells were fixed with freshly prepared 4% v/v paraformaldehyde (#1578100, Electron Microscopy Sciences) for 20 min. Fixed cells were washed 3 times with PBS before permeabilization with 0.3% v/v Triton X-100 (#T8787, Sigma-Aldrich) for 1 h. Triton X-100 was prepared in a solution of 1% w/v BSA (#A3059, Sigma-Aldrich) in PBS. After permeabilization, cells were washed with PBS and incubated in blocking solution (1% w/v BSA in PBS) for 1 h. To visualize endogenous polyP, cells were incubated with PPXBD-mCherry at a concentration of 10 μg/ml prepared in blocking solution overnight at room temperature. To stain P-bodies, a mouse monoclonal antibody against EDC4 (#sc-374211, Santa Cruz Biotechnology) or a rabbit monoclonal antibody against DCP1A (# NBP2-59785, Novus Biologicals) were used at a concentration of 2 μg/ml. To stain SGs, a rabbit polyclonal antibody against G3BP1 (#130572AP, Proteintech) was used at 1 μg/ml. Cells were incubated simultaneously with the PPXBD-mCherry probe and the respective primary antibodies. The next day, cells were washed with 1× PBS and incubated with respective secondary antibodies for 2 h at room temperature protected from light. The secondary antibodies that we used were goat anti-mouse IgG-Alexa Fluor 647 (#A21235, Invitrogen) and goat anti-rabbit IgG-Alexa Fluor 488 (#A11008, Invitrogen). Cells were then washed with 1× PBS and incubated with DAPI (#D1306, Thermo Fisher Scientific) at a concentration of 1 μg/ml for 10 min to stain the nucleus. Cells were washed 3 times before mounting them onto objective slides using Prolong Gold (#9071S, Cell Signaling Technology) as the mounting medium. Mounted coverslips were sealed with nail polish and imaged 24 h post-mounting. Mounted cells were visualized using a 63× oil objective on a Leica SP8 laser scanning confocal microscope (Leica GmbH, Mannheim Germany) on a DMI8 base using the LAS X software.

## Supporting information

S1 Fig PolyP-mediated Hfq foci formation in *Escherichia coli* (supplementing Fig 1). **(A)** Growth (solid lines) and nitrogen (N) levels (dashed lines) of WT, Δ*ppk*, Δ*hfq*, and Δ*hfq*Δ*ppk E. coli* MG1655 in Gutnick minimal medium supplemented with 3 mM NH4Cl. Cells were grown at 37 °C with aeration. Entry of N starvation (N0) is indicated. Error bars indicate SD (*n* = 3). **(B)** Subcellular localization of Hfq-mCherry in N24 MG1655 *hfq::hfq-mCherry* WT or *Δppk* at indicated time points after 10% 1,6-hexanediol treatment. Bacteria containing multiple foci are highlighted by arrowheads. **(C)** Hfq-mCherry levels at N+ or N24 as determined by western blot using mCherry antibody. Total protein was used as loading control. Hfq-mCherry signal in N+ MG1655 *hfq::hfq-mCherry* cells was set to 100%. Comparison was made by two-way ANOVA. Error bars indicate SD (*n* = 3). ***p < 0.001. **(D)** Ratio of mean Hfq intensities in foci, versus non-focus pixels of the corresponding cells, for nitrogen-starved *hfq::hfq-mCherry* cells in the indicated genotypes. Mean values are shown for four imaging fields in each of three biological replicates (points). **(E)** Immunofluorescence images of fixed N24 WT or *Δppk E. coli* MG1655 *hfq::hfq-3xFLAG* using anti-FLAG antibodies for visualization. Hfq foci are indicated by arrowheads. **(F)** Quantification of bacteria containing Hfq-3xFLAG foci from same experiments as shown in (E). **(G)** Bacterial ATP levels normalized to total protein in indicated strains and conditions. ATP levels in WT N+ samples were set to 1. Comparisons were made with two-way ANOVA. Error bars indicate SD (*n* = 3). **p < 0.01. Comparison was made by unpaired *t* test. Error bars indicate SD (*n* = 5). ****p < 0.0001. **(H)** Subcellular localization of Hfq-mCherry in exponentially growing Δ*ppk* or Δ*ppk*Δ*tmaR E. coli hfq::hfq-mCherry* carrying an empty plasmid or a plasmid expressing *ppk10k*. (**I**) Quantification of bacteria containing Hfq-mCherry foci in indicated strains after 4 h of osmotic stress as in Fig 1L. Scale bars: 5 µm. **(J)** PolyP extracted from *E. coli* MG1655 *hfq::hfq-mCherry* after 4 h of mock treatment (control) or upon deep stationary phase (24h), osmotic stress treatment with 300 mM or 1.17 M NaCl in LB medium was visualized on TBE gel by DAPI staining and photobleaching. PolyP was shown as dark smears (arrowhead). Synthetic 300mer polyP was used as a reference. All underlying data can be found in S1 Data.
(TIF)

S2 Fig Schematic of classification of Hfq-PAmCherry trajectories by their overlap with Hfq foci (black dashed circle) and subsequent assignment into three states: inclusion in a slow-diffusing condensate (red), a slow-moving nucleoid-associated state (yellow), and a fast-diffusing free state (blue). (Related to Fig 2).
(TIF)

S3 Fig Representative time course images showing the fluorescence recovery of Hfq-mCherry in indicated strains from the same experiment in Fig 3D. Scale bar: 5 µm. (Related to Fig 3).
(TIF)

S4 Fig Effects of polyP status on transcript stability in the N24 stress condition (related to Fig 5). **(A)** Comparisons of fitted half-lives for transcripts in the WT vs. *ppk* cell; a red line is shown along the main diagonal. **(B)** Full gene set enrichment analysis (performed using iPAGE software) on transcripts discretized into bins matching the categories in Fig 5K. All underlying data can be found in S5 Data.
(TIF)

S5 Fig Manipulation of endogenous polyP levels in mammalian cells (related to Fig 6). **(A)** A stable NIH3T3 cell line expressing a destabilization domain (DD) tagged yPPX was incubated in the absence (uninduced) and presence (induced) of small molecule, Shield1 (0.5 µM) that allows for the expression of yPPX. The cells were fixed and stained for polyP with PPXBD-mCherry. **(B)** PolyP levels were quantified based on fluorescence intensity of the PPXBD-mCherry probe. Each data point represents the average mCherry fluorescence intensity per cell (after background correction) in an image of at least 20 cells. **(C)** NIH3T3 cells were transiently transfected with a bacterial polyP kinase (*Ec*PPK1) and were fixed and stained for polyP with PPXBD-mCherry. **(D)** PolyP levels were quantified based on fluorescence intensity of the PPXBD-mCherry probe. Each data point represents the mCherry fluorescence intensity of one cell (after background correction). An unpaired *t* test was used to assess statistical significance (B, D). A representative set of images and quantification is shown (*n* = 3). Scale bars: 10 µm. All underlying data can be found in S6 Data.
(TIF)

S1 Table (Related to Fig 5): protein composition of Hfq HMW complexes. (DOC)

S2 Table (Related to Fig 5): GO-term enrichment analysis of Hfq HMW complexes. (DOC)

S3 Table Strains and plasmids used in this study. (DOC)

S1 Raw Images Uncropped and annotated images of Figs 1A, 1G, 2D, 4D with AF647 (upper left), FAM (upper right) and Coomassie channels (lower left), 4E, 4F with anti-mCherry (left) and DAPI (right) channels; the left panel is also used in Fig 5A for method illustration purposes; 4G including anti-mCherry (left) and anti-GFP channels (left); S1J Fig. (PDF)

S1 Data Data underlying Fig panels 1A, 1C, 1E, 1F, 1I, S1A, S1C, S1D, S1F, S1G, and S1I. (XLSX)

S2 Data Data underlying Fig panels 2A, 2C, and 2E–2H. (XLSX)

S3 Data Data underlying Fig panels 3B–3E. (XLSX)

S4 Data Data underlying Fig panels 4B and 4I. (XLSX)

S5 Data Data underlying Fig panels 5F, 5I, S4A, and S4B. (XLSX)

S6 Data Data underlying Fig panels 6E, 6G, S5B, and S5D. (XLSX)

S7 Data Mass spectrometry results used for generating S1 Table and overlaps of GO terms annotated to proteins identified in the Hfq/polyP foci vs. those in human P-bodies and stress granules. In the supplementary data file, the first tab gives more detailed descriptions of the data sets shown and the analysis used.
(XLSX)

S8 Data Mass spectrometry results of Hfq pulldowns. The data file contains normalized protein abundances arising from MS/MS on Hfq pulldowns in the indicated conditions and was used in identifying the detected proteins for the analysis presented in the text.
(XLSX)

S9 Data Fitted values and comparisons obtained from all high-throughput sequencing experiments used in this study. In the supplementary data file, the first tab gives more detailed descriptions of the data sets shown, and the second tab gives the fitted values and other statistics used in our analysis.
(XLSX)

## Abbreviations

EMSA: electrophoretic mobility shift assay
FISH: fluorescence in situ hybridization
FRAP: fluorescence recovery after photobleaching
GO: gene ontology
HMW: high molecular weight
IFs: initiation factors
LLPS: liquid–liquid phase separation
LMW: low molecular weight
MAD: median absolute deviation
PBS: phosphate-buffered saline
SGs: stress granules
TBE: tris-borate EDTA
