# Supplementary figures and images for: Polyphosphate modulates the stress-responsive formation of functional RNA-protein condensates in bacteria and mammalian cells
Source: PLoS Biol. 2026 Apr 27;24(4):e3003775. doi: 10.1371/journal.pbio.3003775 (PMC13193609; doi:10.1371/journal.pbio.3003775)

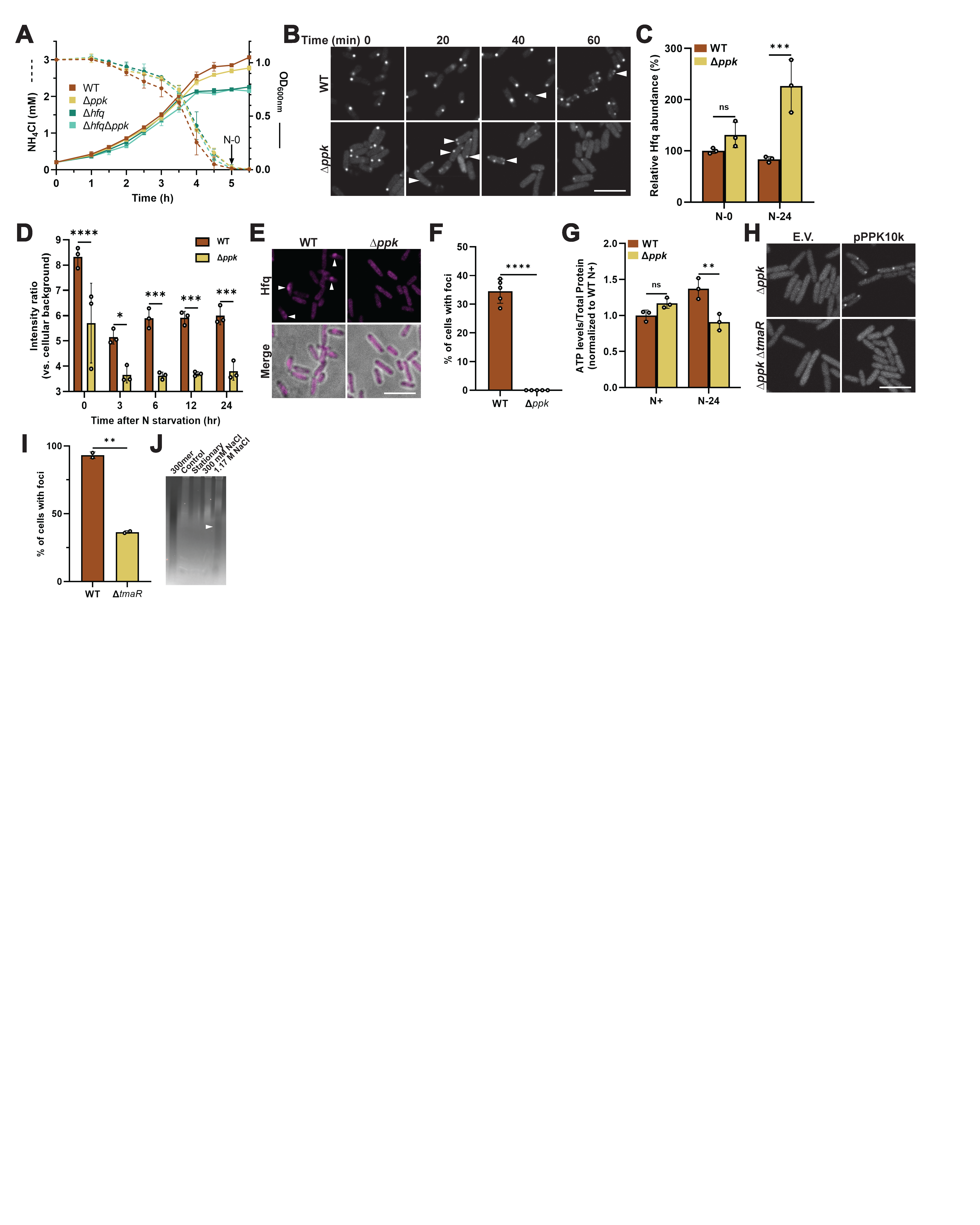

Supplement: S1 Fig — (A) Growth (solid lines) and nitrogen (N) levels (dashed lines) of WT, Δppk, Δhfq, and ΔhfqΔppk E. coli MG1655 in Gutnick minimal medium supplemented with 3 mM NH4Cl. Cells were grown at 37 °C with aeration. Entry of N starvation (N0) is indicated. Error bars indicate SD (n = 3). (B) Subcellular localization of Hfq-mCherry in N24 MG1655 hfq::hfq-mCherry WT or Δppk at indicated time points after 10% 1,6-hexanediol treatment. Bacteria containing multiple foci are highlighted by arrowheads. (C) Hfq-mCherry levels at N+ or N24 as determined by western blot using mCherry antibody. Total protein was used as loading control. Hfq-mCherry signal in N+ MG1655 hfq::hfq-mCherry cells was set to 100%. Comparison was made by two-way ANOVA. Error bars indicate SD (n = 3). ***p < 0.001. (D) Ratio of mean Hfq intensities in foci, versus non-focus pixels of the corresponding cells, for nitrogen-starved hfq::hfq-mCherry cells in the indicated genotypes. Mean values are shown for four imaging fields in each of three biological replicates (points). (E) Immunofluorescence images of fixed N24 WT or Δppk E. coli MG1655 hfq::hfq-3xFLAG using anti-FLAG antibodies for visualization. Hfq foci are indicated by arrowheads. (F) Quantification of bacteria containing Hfq-3xFLAG foci from same experiments as shown in (E). (G) Bacterial ATP levels normalized to total protein in indicated strains and conditions. ATP levels in WT N+ samples were set to 1. Comparisons were made with two-way ANOVA. Error bars indicate SD (n = 3). **p < 0.01. Comparison was made by unpaired t test. Error bars indicate SD (n = 5). ****p < 0.0001. (H) Subcellular localization of Hfq-mCherry in exponentially growing Δppk or ΔppkΔtmaR E. coli hfq::hfq-mCherry carrying an empty plasmid or a plasmid expressing ppk10k. (I) Quantification of bacteria containing Hfq-mCherry foci in indicated strains after 4 h of osmotic stress as in Fig 1L. Scale bars: 5 µm. (J) PolyP extracted from E. coli MG1655 hfq::hfq-mCherry after 4 h of moc [file pbio.3003775.s001.tif]

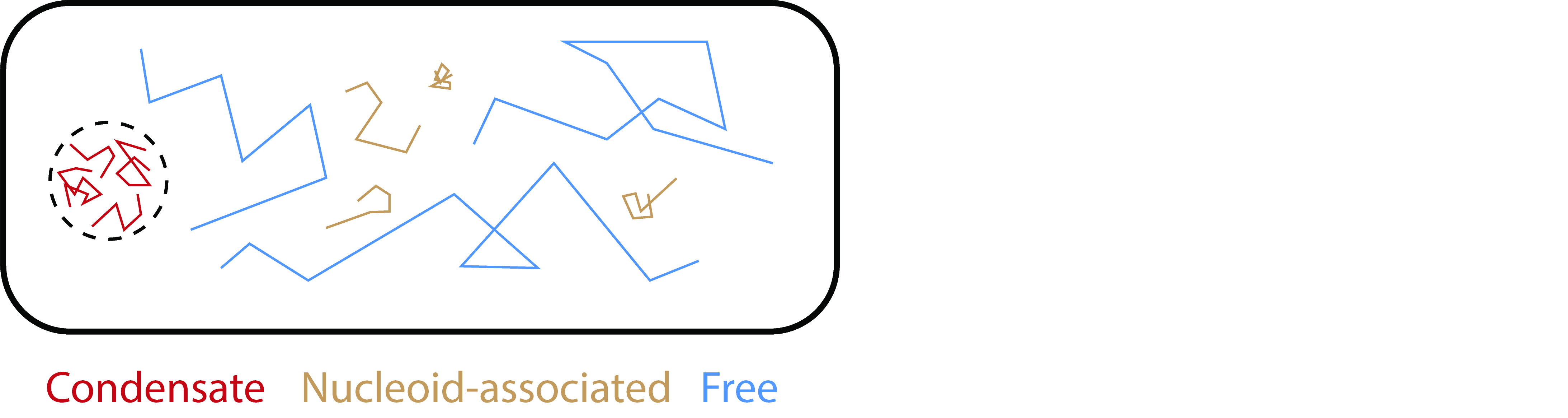

Supplement: S2 Fig — (Related to Fig 2). (TIF) [file pbio.3003775.s002.tif]

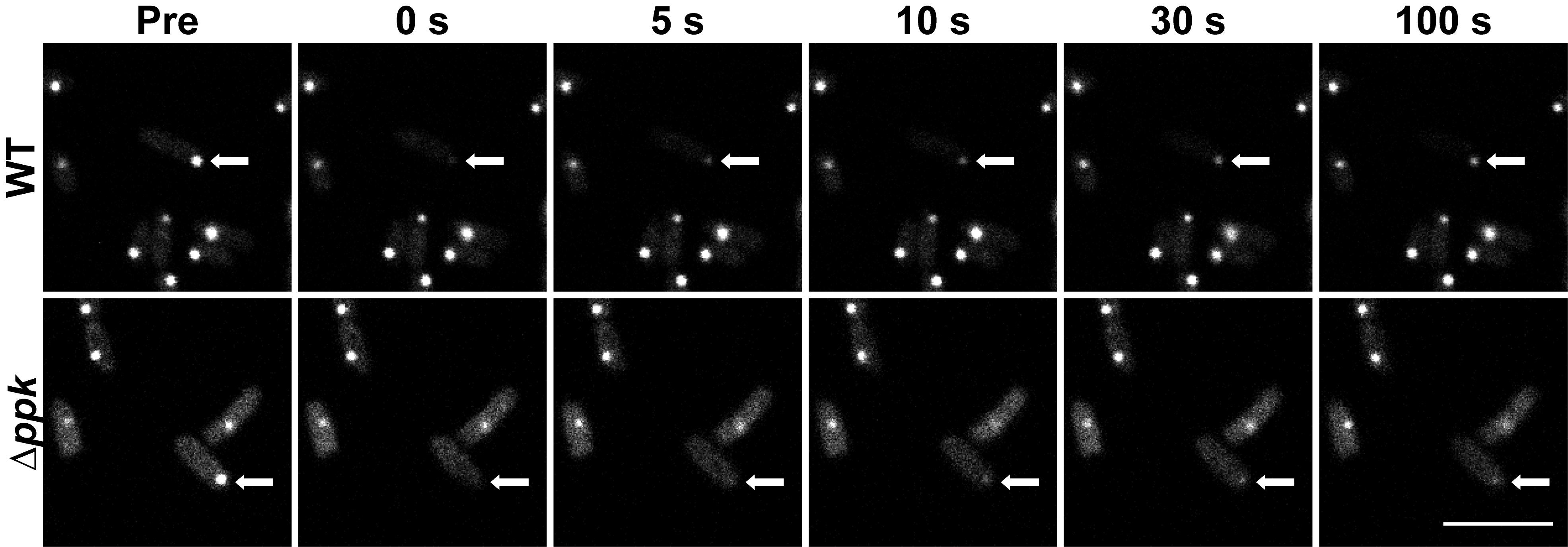

Supplement: S3 Fig — Scale bar: 5 µm. (Related to Fig 3). (TIF) [file pbio.3003775.s003.tif]

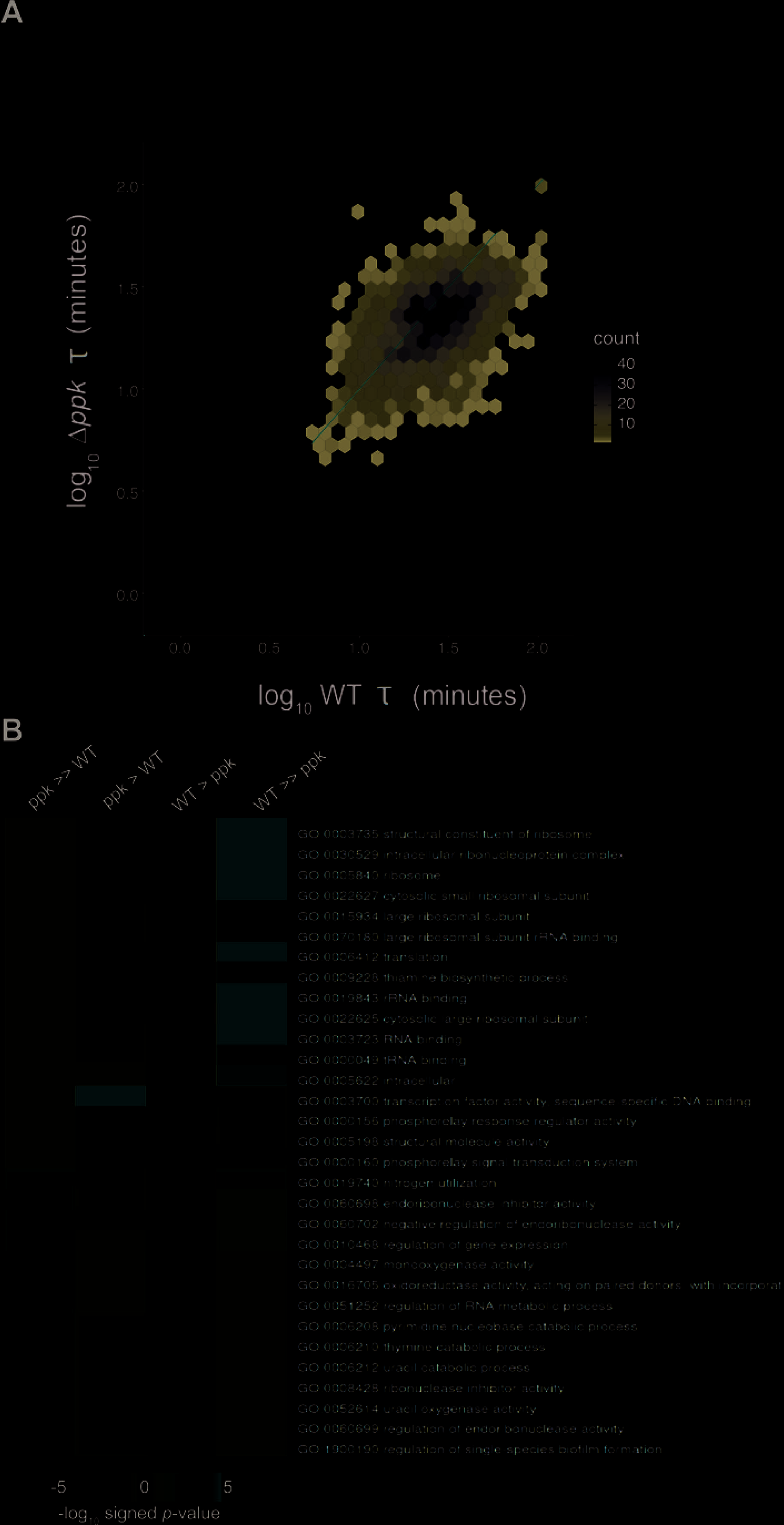

Supplement: S4 Fig — (A) Comparisons of fitted half-lives for transcripts in the WT vs. ppk cell; a red line is shown along the main diagonal. (B) Full gene set enrichment analysis (performed using iPAGE software) on transcripts discretized into bins matching the categories in Fig 5K. All underlying data can be found in S5 Data. (TIF) [file pbio.3003775.s004.tif]

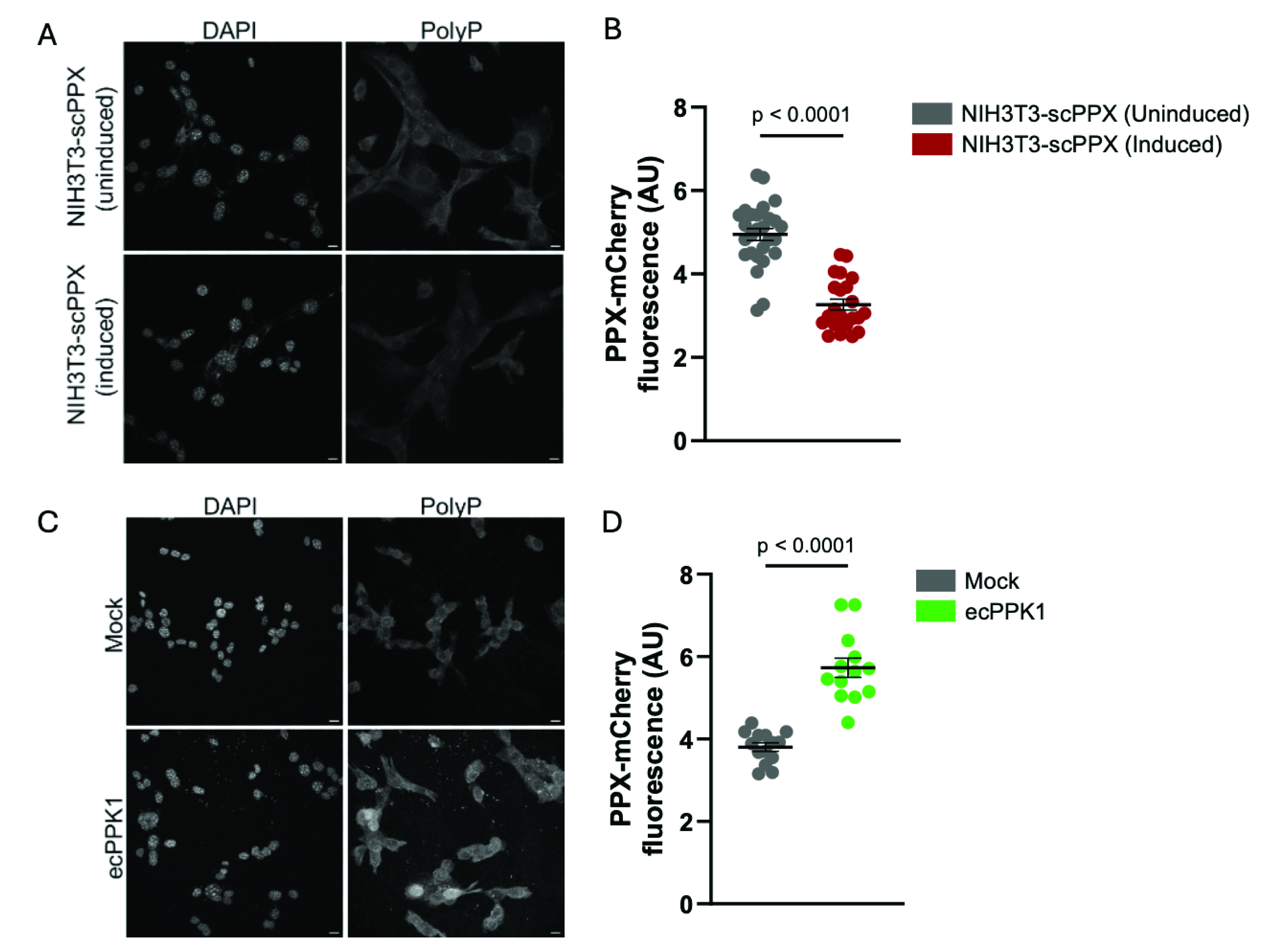

Supplement: S5 Fig — (A) A stable NIH3T3 cell line expressing a destabilization domain (DD) tagged yPPX was incubated in the absence (uninduced) and presence (induced) of small molecule, Shield1 (0.5 µM) that allows for the expression of yPPX. The cells were fixed and stained for polyP with PPXBD-mCherry. (B) PolyP levels were quantified based on fluorescence intensity of the PPXBD-mCherry probe. Each data point represents the average mCherry fluorescence intensity per cell (after background correction) in an image of at least 20 cells. (C) NIH3T3 cells were transiently transfected with a bacterial polyP kinase (EcPPK1) and were fixed and stained for polyP with PPXBD-mCherry. (D) PolyP levels were quantified based on fluorescence intensity of the PPXBD-mCherry probe. Each data point represents the mCherry fluorescence intensity of one cell (after background correction). An unpaired t test was used to assess statistical significance (B, D). A representative set of images and quantification is shown (n = 3). Scale bars: 10 µm. All underlying data can be found in S6 Data. (TIF) [file pbio.3003775.s005.tif]
