# Supplementary material for: Polyphosphate modulates the stress-responsive formation of functional RNA-protein condensates in bacteria and mammalian cells
Source: PLoS Biol. 2026 Apr 27;24(4):e3003775. doi: 10.1371/journal.pbio.3003775 (PMC13193609; doi:10.1371/journal.pbio.3003775)
Supplement: S1 Table — (DOC) [file pbio.3003775.s006.doc]

**Table S1:** **Proteins enriched in Hfq-HMW complexes (related to Figure 5, Supplementary data set 2).** Lysates were prepared from N24 *hfq::hfq-*mCherry WT or the *hfq::hfq-*mCherry Δ*ppk* strain and analyzed on native PAGE. In addition, N24 *hfq::hfq-*mCherry WT lysate was treated with either yPPX to degrade polyP or RNaseA to degrade RNA prior to the native PAGE. Corresponding regions of the gels were excised, proteins pairwise differentially labelled (WT v *ppk*; WT v WT + yPPX; WT v WT + RNaseA) and analyzed by MS/MS analysis. Proteins with a log2 fold change (l2fc) >1 in at least 3 out of 5 replicates in WT v Δ*ppk*, 2 out of 4 replicates in WT v WT + yPPX or 2 out of 2 in WT v WT + RNase A are shown.

| **Acc #** | **Gene** | **Description** | **Enriched in WT lysate**  ***versus***  **∆*ppk* + yPPX + RNaseA** | | |
| --- | --- | --- | --- | --- | --- |
| C4ZR49 | **Hfq** | **RNA-binding protein** | **X** |  |  |
| P0A7B1 | Ppk | Polyphosphate kinase | **X** | **X** |  |
| P0AG20 | RelA | GTP pyrophosphokinase | **X** |  |  |
| B1XAY9 | HisS | Histidine--tRNA ligase | **X** | **X** |  |
| B1XGY0 | InfB | Translation initiation factor IF-2 | **X** | **X** | **X** |
| C4ZYI0 | InfC | Translation initiation factor IF-3 | **X** | **X** |  |
| C4ZZ48 | RhlB | ATP-dependent RNA helicase | **X** | **X** |  |
| P37765 | RluB | Ribosomal large subunit pseudouridine synthase B | **X** | **X** |  |
| P33643 | RluD | Ribosomal large subunit pseudouridine synthase D | **X** | **X** |  |
| C4ZQ93 | RmlL | Ribosomal RNA large subunit methyltransferase I | **X** |  |  |
| P21338 | Rna | Ribonuclease I | **X** |  |  |
| B1XDV3 | RplL | 50S ribosomal protein L9 | **X** | **X** |  |
| B1X6E6 | RplQ | 50S ribosomal protein L17 | **X** | **X** |  |
| C4ZUG4 | RplX | 50S ribosomal protein L24 | **X** | **X** |  |
| P0AG51 | RpmD | 50S ribosomal protein L30 | **X** |  |  |
| P0A7W1 | RpsE | 30S ribosomal protein S5 | **X** | **X** |  |
| P0ADZ4 | RpsO | 30S ribosomal protein S15 | **X** |  |  |
| P0A7T3 | RpsP | 30S ribosomal protein S16 | **X** | **X** |  |
| P0A7U3 | RpsS | 30S ribosomal protein S19 | **X** | **X** |  |
| P60390 | RsmH | Ribosomal RNA small subunit methyltransferase H | **X** | **X** |  |
| P14081 | SelB | Selenocysteine-specific elongation factor | **X** |  |  |
| B1XBU0 | SmpB | SsrA-binding protein | **X** |  |  |
| P00954 | TrpS | Tryptophan--tRNA ligase | **X** | **X** |  |
| B1XFU9 | TyrS | Tyrosine--tRNA ligase | **X** | **X** |  |
| P0AGJ5 | YfiF | Uncharacterized tRNA/rRNA methyltransferase | **X** | **X** |  |
| C4ZX86 | Der | GTPase Der | **X** | **X** |  |
| P0AEH5 | ElaB | Protein ElaB | **X** |  |  |
| B1XBZ0 | RpoC | DNA-directed RNA polymerase subunit beta' | **X** |  |  |
| B1X8V5 | CbpA | Curved DNA-binding protein | **X** | **X** |  |
| P0A972 | CspE | Cold shock-like protein | **X** | **X** |  |
| P0AES6 | GyrB | DNA gyrase subunit B | **X** | **X** |  |
| P0ACF0 | HupA | DNA-binding protein HU-alpha | **X** | **X** |  |
| P0ACF4 | HupB | DNA-binding protein HU-beta | **X** | **X** |  |
| C4ZQE5 | RvuA | Holliday junction ATP-dependent DNA helicase | **X** | **X** |  |
| P06612 | TopA | DNA topoisomerase 1 | **X** | **X** |  |
| P0A698 | UvrA | UvrABC system protein A | **X** | **X** |  |
| P0A6Y8 | DnaK | Chaperone protein DnaK | **X** |  |  |
| P0A9M0 | Lon | Lon protease | **X** | **X** |  |
| P0AEU7 | Skp | Chaperone protein Skp | **X** | **X** |  |
| B1X927 | AccD | Acetyl-coenzyme A carboxyl transferase sub B | **X** |  |  |
| B1XDI9 | Eno | Enolase | **X** | **X** |  |
| P0A991 | FbaB | Fructose-bisphosphate aldolase class 1 | **X** | **X** |  |
| P0A9B2 | GapA | Glyceraldehyde-3-phosphate dehydrogenase A | **X** | **X** | **X** |
| P15877 | Gcd | Quinoprotein glucose dehydrogenase | **X** | **X** |  |
| P0A6V1 | GlgC | Glucose-1-phosphate adenylyltransferase | **X** | **X** | **X** |
| P21599 | PykA | Pyruvate kinase II | **X** | **X** |  |
| P0AD61 | PykF | Pyruvate kinase I | **X** | **X** | **X** |
| P0ABQ0 | CoaBC | Coenzyme A biosynthesis bifunctional protein | **X** |  |  |
| P38038 | CysJ | Sulfite reductase [NADPH] flavoprotein alpha-comp | **X** | **X** | **X** |
| P0AA89 | DosC | Diguanylate cyclase | **X** |  |  |
| P0A6P7 | EngB | Probable GTP-binding protein | **X** | **X** |  |
| P39180 | Flu | Antigen 43 | **X** | **X** |  |
| P63235 | GadC | Glutamate/gamma-aminobutyrate antiporter | **X** | **X** |  |
| P0ACC7 | GlmU | Bifunctional protein | **X** | **X** |  |
| P09831 | GltB | Glutamate synthase [NADPH] large chain | **X** | **X** | **X** |
| P0ADG7 | GuaB | Inosine-5'-monophosphate dehydrogenase | **X** | **X** |  |
| P06987 | HisB | Histidine biosynthesis bifunctional protein HisB | **X** |  |  |
| C4ZXA5 | IscS | Cysteine desulfurase IscS | **X** | **X** |  |
| C4ZRS3 | LpxA | Acyl- UDP-N-acetylglucosamine O-acyltransferase | **X** | **X** |  |
| P21645 | LpxD | UDP-3-O-(3-hydroxymyristoyl)glucosamine N-acyltransferase | **X** | **X** |  |
| P45955 | CpoB | Cell division coordinator | **X** | **X** |  |
| P0AEZ3 | MinD | Septum site-determining protein | **X** | **X** |  |
| C4ZXV5 | MoaC | Cyclic pyranopterin monophosphate synthase | **X** | **X** |  |
| P02931 | OmpF | Outer membrane porin F | **X** | **X** |  |
| P76002 | PliG | Inhibitor of g-type lysozyme | **X** | **X** |  |
| P23865 | Prc | Tail-specific protease | **X** | **X** |  |
| P0AGC3 | Slt | Soluble lytic murein transglycosylase | **X** | **X** |  |
| C4ZRQ6 | YaeH | UPF0325 protein | **X** | **X** |  |
| P75863 | YbcbX | Uncharacterized protein | **X** | **X** |  |
| P0A9K3 | YbeZ | PhoH-like protein | **X** | **X** |  |
| P30177 | YbiB | Uncharacterized protein | **X** | **X** |  |
| P76177 | YdgH | Protein YdgH | **X** | **X** |  |
| P0ACY1 | YdjA | Putative NAD(P)H nitroreductase | **X** | **X** |  |
| P46853 | YhhX | Uncharacterized oxidoreductase | **X** | **X** |  |
| P76116 | YncE | Uncharacterized protein YncE | **X** | **X** |  |
