# Supplementary material for: Polyphosphate modulates the stress-responsive formation of functional RNA-protein condensates in bacteria and mammalian cells
Source: PLoS Biol. 2026 Apr 27;24(4):e3003775. doi: 10.1371/journal.pbio.3003775 (PMC13193609; doi:10.1371/journal.pbio.3003775)
Supplement: S2 Table — (DOC) [file pbio.3003775.s007.doc]

**Table S2: GO-term enrichment analysis of Hfq-foci associated proteins (related to Fig. 6A).** Shown are the set of GO terms present in the three-way interface between Hfq-foci, human P-bodies, and human stress granules.

| DNA-related |
| --- |
| GO:0003677 DNA binding |
| GO:0003697 single-stranded DNA binding |
| GO:0006281 DNA repair |
| GO:0006355 regulation of transcription, DNA-templated |
| RNA-related |
| GO:0003723 RNA binding |
| GO:0003724 RNA helicase activity |
| GO:0006401 RNA catabolic process |
| GO:0019843 rRNA binding |
| GO:0008298 intracellular mRNA localization |
| GO:1990904 ribonucleoprotein complex |
| GO:0008143 poly(A) binding |
| GO:0003676 nucleic acid binding |
| Translation related |
| GO:0003743 translation initiation factor activity |
| GO:0005840 ribosome |
| GO:0006412 translation |
| GO:0006417 regulation of translation |
| GO:0043022 ribosome binding |
| Stress related |
| GO:0009410 response to xenobiotic stimulus |
| GO:0009636 response to toxic substance |
| GO:0034605 cellular response to heat |
| GO:0042594 response to starvation |
| Locations |
| GO:0005576 extracellular region |
| GO:0005694 chromosome |
| GO:0005737 cytoplasm |
| GO:0005829 cytosol |
| GO:0016020 membrane |
| GO:0005886 plasma membrane |
| Other |
| GO:0000287 magnesium ion binding |
| GO:0005524 ATP binding |
| GO:0007165 signal transduction |
| GO:0008270 zinc ion binding |
| GO:0010468 regulation of gene expression |
| GO:0015031 protein transport |
| GO:0016301 kinase activity |
| GO:0016887 ATPase activity |
| GO:0042802 identical protein binding |
| GO:0042803 protein homodimerization activity |
| GO:0046872 metal ion binding |
| GO:0065003 protein-containing complex assembly |
| GO:0032991 protein-containing complex |
