# Supplementary material for: Polyphosphate modulates the stress-responsive formation of functional RNA-protein condensates in bacteria and mammalian cells
Source: PLoS Biol. 2026 Apr 27;24(4):e3003775. doi: 10.1371/journal.pbio.3003775 (PMC13193609; doi:10.1371/journal.pbio.3003775)
Supplement: S3 Table — (DOC) [file pbio.3003775.s008.doc]

**Table S3. Strains, plasmids and cell lines used in this study.**

| **Bacterial strain** | | **Marker(s)** | **Source** |
| --- | --- | --- | --- |
| BL21 (DE3) *(F- ompT gal dcm lon hsdSB(rB- mB-) λ(DE3[lacI lacUV5-T7 gene 1 ind1 sam7 nin5]) ykgD:: cat+ )* | |  | Lab stock |
| BL21(DE3) pET21a-*hfq* | | Amp | [1] |
| BL21(DE3) pET21a-*hfq*S65C | | Amp | [1] |
| MG1655 (*F-, λ-, rph-1 ilvG- rfb-50*) | |  | [2] |
| MG1655 *Δppk* | |  | [1] |
| MG1655 *Δhfq* | |  | [1] |
| MG1655 *ΔhfqΔppk* | |  | [1] |
| MG1655 *Δppx* | |  | [3] |
| MG1655 *Δppk* pBAD18b | | Amp | [1] |
| MG1655 *Δppk* pBAD18b *ppk* | | Amp | [1] |
| MG1655 *Δppk* pBAD18b *hfq* | | Amp | [1] |
| MG1655 *Δhfq* pBAD18b | | Amp | [1] |
| MG1655 *Δhfq* pBAD18b *ppk* | | Amp | [1] |
| MG1655 *Δhfq* pBAD18b *hfq* | | Amp | [1] |
| MG1655 *hfq::hfq-PAmcherry* | |  | [1] |
| MG1655 *hfq::hfq-PAmcherry Δppk* | |  | [1] |
| MG1655 *hfq::hfq-mCherry* | |  | This study |
| MG1655 *hfq::hfq-mcherry Δppk* | |  | This study |
| MG1655 *hfq::hfq-mcherry Δppx* | |  | This study |
| MG1655 *hfq::hfq*-*3xFLAG* | |  |  |
| MG1655 *hfq::hfq*-*3xFLAG* *Δppk* | |  |  |
| MG1655 *hfq::hfq-mcherry* Δ*tmaR* | |  | This study |
| MG1655 *hfq::hfq-mcherry* Δ*tmaR* Δ*ppk* | |  | This study |
| MG1655 *hfq::hfq-mcherry* Δ*tmaR* pTmaR-mNeonGreen | | Kan | This study |
| MG1655 *hfq::hfq-mcherry* Δ*tmaR* Δ*ppk* pTmaR-mNeonGreen | | Kan | This study |
| MG1655 Δ*hfq* Δ*tmaR* pTmaR-mNeonGreen | | Kan | This study |
| MG1655 Δ*hfq* Δ*ppk* Δ*tmaR* pTmaR-mNeonGreen | | Kan | This study |
| MG1655 *hfq::hfq-mCherry rne::rne-mTurquoise2* | |  | This study |
| MG1655 *hfq::hfq-mCherry rhlB::rhlB-mTurquoise2* | |  | This study |
| MG1655 *hfq::hfq-mCherry pnp::pnp-mTurquoise2* | |  | This study |
| MG1655 *hfq::hfq-mCherry eno::eno-mTurquoise2* | |  | This study |
| MG1655 *hfq::hfq-mCherry rne::rne-mTurquoise2 Δppk* | |  | This study |
| MG1655 *hfq::hfq-mCherry rhlB::rhlB-mTurquoise2 Δppk* | |  | This study |
| MG1655 *hfq::hfq-mCherry pnp::pnp-mTurquoise2 Δppk* | |  | This study |
| MG1655 *hfq::hfq-mCherry eno::eno-mTurquoise2 Δppk* | |  | This study |
| MG1655 *hfq::hfq-mcherry* Δ*ppk* pBAD18b | | Amp | This study |
| MG1655 *hfq::hfq-mcherry* Δ*ppk* pBAD18b *ppk* | | Amp | This study |
| MG1655 *hfq::hfq-mcherry* Δ*ppk* pBAD18b *hfq* | | Amp | This study |
| MG1655 *hfq::hfq-mCherry Δppk* pWSK129 | | Kan | This study |
| MG1655 *hfq::hfq-mCherry Δppk* pPPK | | Kan | This study |
| MG1655 *hfq::hfq-mCherry Δppk* pPPK10k | | Kan | This study |
| MG1655 *hfq::hfq-mCherry Δppk ΔtmaR* pWSK129 | | Kan | This study |
| MG1655 *hfq::hfq-mCherry Δppk ΔtmaR* pPPK10k | | Kan | This study |
| **Mammalian cell line** | |  | |
| HEK293 | | ATCC® CRL-1573™ | |
| HeLa | | ATCC® CCL-2™ | |
| NIH3T3 | | ATCC® CRL-1648™ | |
| **Plasmid** | **Description** | **Markers** | **Source** |
| pKD4 | Kanamycin resistance cassette donor | Kan | [4] |
| pKD46 | λ red recombinase | Amp | [4] |
| pCP20 | Flp recombinase | Amp | [4] |
| pET21a | IPTG inducible vector for protein purification | Amp | Novagen |
| pET21a-*hfq* | IPTG inducible vector for Hfq purification | Amp | [1] |
| pET21a-*hfqS65C* | IPTG inducible vector for Hfq S65C purification | Amp | [1] |
| pWSK129 | Low copy number vector | Kan | [5] |
| pPPK | Expression vector for *ppk* under endogenous *ppk* promoter based on pWSK129 | Kan | [5] |
| pPPK10k | Expression vector for *ppk10k* under endogenous *ppk* promoter based on pWSK129 | Kan | [5] |
| pTmaR-mNeonGreen | Expression vector for *tmaR-mNG* under endogenous *tmaR* promoter based on pWSK129 | Kan | This study |
| pBAD18b | Arabinose-inducible expression vector | Amp | [1] |
| pBAD18b *ppk* | Arabinose-inducible expression vector for *ppk* | Amp | [1] |
| pBAD18b *hfq* | Arabinose-inducible expression vector for *hfq* | Amp | [1] |
| pcDNA3.1(-) | Mammalian expression vector | Amp | [6] |
| *Ec*PPK1 | Mammalian expression vector for *E. coli* PPK | Amp | [6] |
| pLVX-pTuner Green | Mammalian expression vector for DD-tagged proteins | Amp | Takara |
| DD-3x-FLAG-scPPX | Mammalian expression vector for DD-tagged yeast PPX | Amp | This study |
| **Oligonucleotide** | **Sequence** | | |
| FAM-rA_30_ | FAM-(rA)_30_ | | |
| hfq-mFP-in-F | GCAACAGGACAGCGAAGAAACCGAATCGGCTGGCTCCGCTGCTGG | | |
| hfq-mFP-in-R | CCGTGTAAAAAAACAGCCCGAAACCCAAGCTTATGAATATCCTCCTTAGTTC | | |
| hfq-mFP-out-F | GTAGCAGCGCGCAGAATACTTCCGCGCAACAGGACAGCGAAGAAACCGAA | | |
| hfq-mFP-out-F | CGGGGAACGCAGGATCGCTGGCTCCCCGTGTAAAAAAACAGCCCGAAACC | | |
| hfq-FLAG-in-F | GCAACAGGACAGCGAAGAAACCGAAGACTACAAAGACCATGACGG | | |
| hfq-FLAG-in-R | CCGTGTAAAAAAACAGCCCGAAACCCCTCCTTAGTTCCTATTCCG | | |
| hfq-seq-F | ATGGCTAAGGGGCAATCTTTACAAG | | |
| rne-mFP-in-F | TCCTGCGCGTCCGCAACCTGTTGAGTCGGCTGGCTCCGCTGCTGG | | |
| rne-mFP-in-R | CGGCAACACATCATGCCTCTGCCGCTCCTGCGCGTCCGCAAC | | |
| rne-mFP-out-F | GGGCTTGATTACTTTGAGCTAATTACAAGCTTATGAATATCCTCCTTAGTTC | | |
| rne-mFP-out-F | AATAAAAAAGCCCTGGCAGTTACCAGGGCTTGATTACTTTGAGCTAATTAC | | |
| rne-seq-F | TTGAAACGGTAGCTGCGGTC | | |
| rhlB-mFP-in-F | TCCGCGTAATCGTCGTCGTTCAGGTTCGGCTGGCTCCGCTGCTGG | | |
| rhlB-mFP-in-R | TGATTTTGAGTATGACATTTTTTATCAAGCTTATGAATATCCTCCTTAGTTC | | |
| rhlB-mFP-out-F | GCAATGGTCCGCGTCGTACTGGCGCTCCGCGTAATCGTCGTCGTTC | | |
| rhlB-mFP-out-F | TCTTGCCATCTTGATACAGTTTGAATGATTTTGAGTATGACATTTTTTATCAAGC | | |
| rhlB-seq-F | ATTGGTCGTACAGGTCGCG | | |
| eno-mFP-in-F | TCGTAAAGAGATCAAAGGCCAGGCATCGGCTGGCTCCGCTGCTGG | | |
| eno-mFP-in-R | ATTTTTAAATCAGATAAAGTCAGTCCAAGCTTATGAATATCCTCCTTAGTTC | | |
| eno-mFP-out-F | TGGGCGAAAAAGCACCGTACAACGGTCGTAAAGAGATCAAAGGCCAGG | | |
| eno-mFP-out-F | AAAAAATGCCAGCCCGGAGGCTGGCATTTTTAAATCAGATAAAGTCAGTCCAAGC | | |
| eno-seq-F | CTGCAATCAAGATGGCGAAAGATG | | |
| mFP-seq-R | GCCCAGTAGCTGACATTCATC | | |
| tmaR-BamHI-220-F | ACACACGGATCCTCAGCTTGAGTTGCTATCGAATCTG | | |
| tmaR-R | CTTCGCTTCGCCGTTTTTCATTTCGC | | |
| tmaR-mFP-in-F | CGAAATGAAAAACGGCGAAGCGAAGTCGGCTGGCTCCGCTGCTG | | |
| mNG-stop-HindIII-R | ACACACAAGCTTTTACTTGTATAATTCGTCCATCCCCATC | | |
| tmaR-mFP-in-R | CAACCCTCATTGAATAAAACGGGAACAAGCTTATGAATATCCTCCTTAGTTC | | |
| tmaR-KO-F | TAAAAGCATTGTCATCTGCGG | | |
| tmaR-KO-R | GCATACAGAAGAAGGCTGCC | | |

**References**

1. Beaufay F, Amemiya HM, Guan J, Basalla J, Meinen B, Chen ZY, Mitral R, Bardwell JCA, Biteen JS, Vecchiarelli AG, Freddolino PL, Jakob U. Polyphosphate drives bacterial heterochromatin formation. Sci Adv. 2021;7(52).

2. Blattner FR, Plunkett G, Bloch CA, Perna NT, Burland V, Riley M, ColladoVides J, Glasner JD, Rode CK, Mayhew GF, Gregor J, Davis NW, Kirkpatrick HA, Goeden MA, Rose DJ, Mau B, Shao Y. The complete genome sequence of Escherichia coli K-12. Science. 1997;277(5331):1453.

3. Gray MJ, Wholey WY, Wagner NO, Cremers CM, Mueller-Schickert A, Hock NT, Krieger AG, Smith EM, Bender RA, Bardwell JCA, Jakob U. Polyphosphate Is a Primordial Chaperone. Mol Cell. 2014;53(5):689–99.

4. Datsenko KA, Wanner BL. One-step inactivation of chromosomal genes in Escherichia coli K-12 using PCR products. P Natl Acad Sci USA. 2000;97(12):6640–5.

5. Rudat AK, Pokhrel A, Green TJ, Gray MJ. Mutations in Escherichia coli Polyphosphate Kinase That Lead to Dramatically Increased In Vivo Polyphosphate Levels. J Bacteriol. 2018;200(6).

6. Bentley-DeSousa A, Holinier C, Moteshareie H, Tseng YC, Kajjo S, Nwosu C, Amodeo GF, Bondy-Chorney E, Sai Y, Rudner A, Golshani A, Davey NE, Downey M. A Screen for Candidate Targets of Lysine Polyphosphorylation Uncovers a Conserved Network Implicated in Ribosome Biogenesis. Cell Reports. 2018;22(13):3427–39.
