# Supplementary material for: Polyphosphate modulates the stress-responsive formation of functional RNA-protein condensates in bacteria and mammalian cells
Source: PLoS Biol. 2026 Apr 27;24(4):e3003775. doi: 10.1371/journal.pbio.3003775 (PMC13193609; doi:10.1371/journal.pbio.3003775)

1A

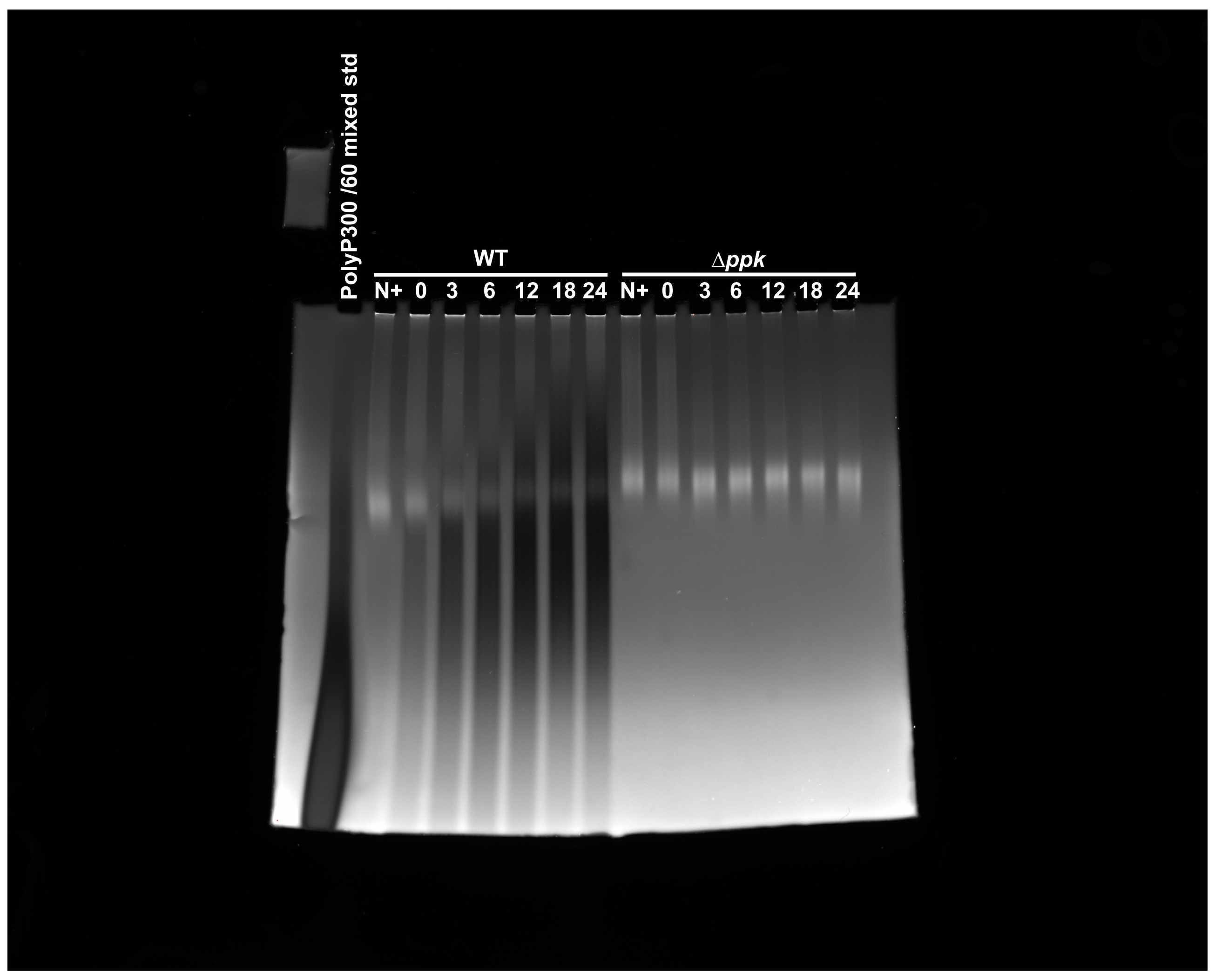

1G

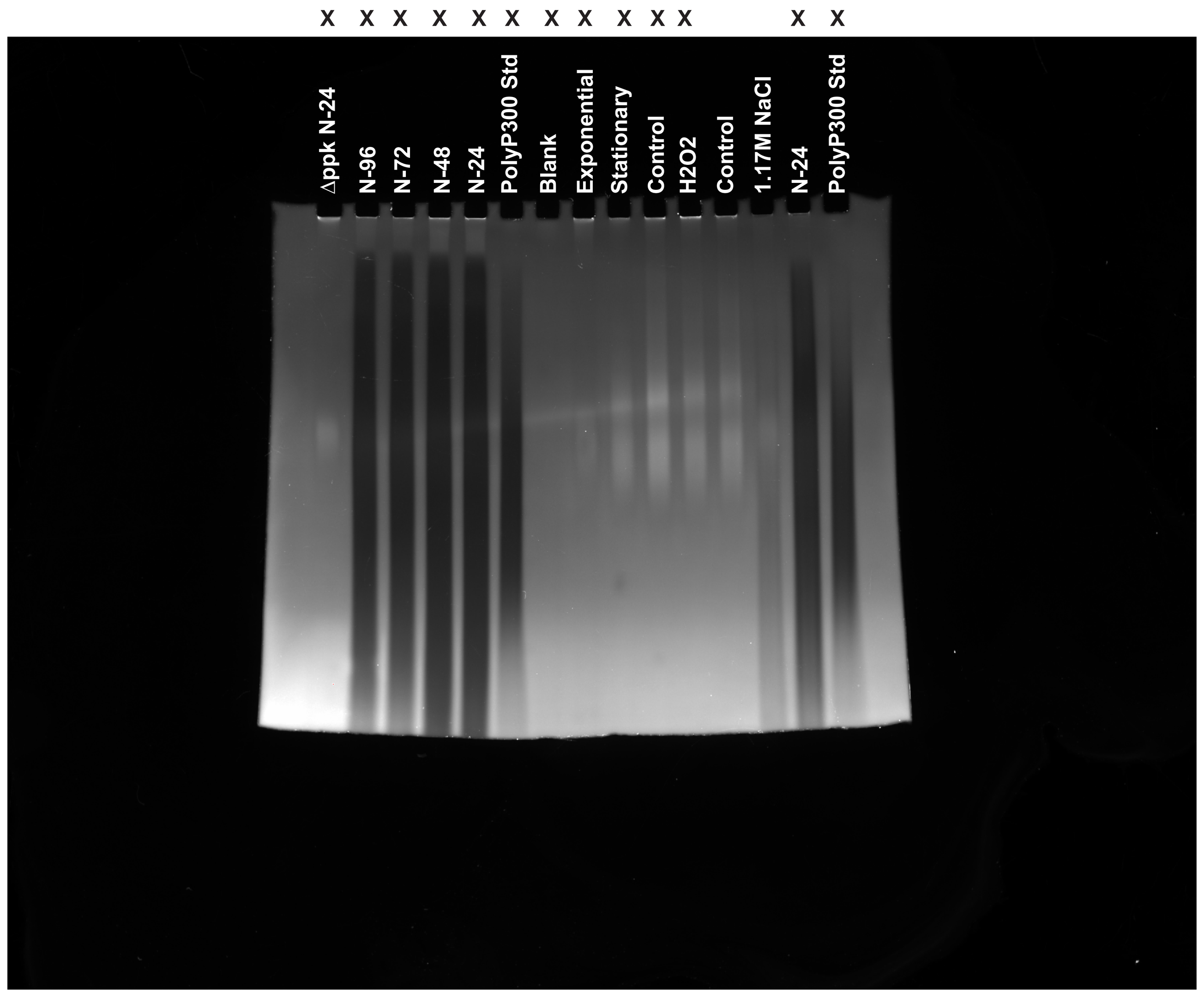

S1J

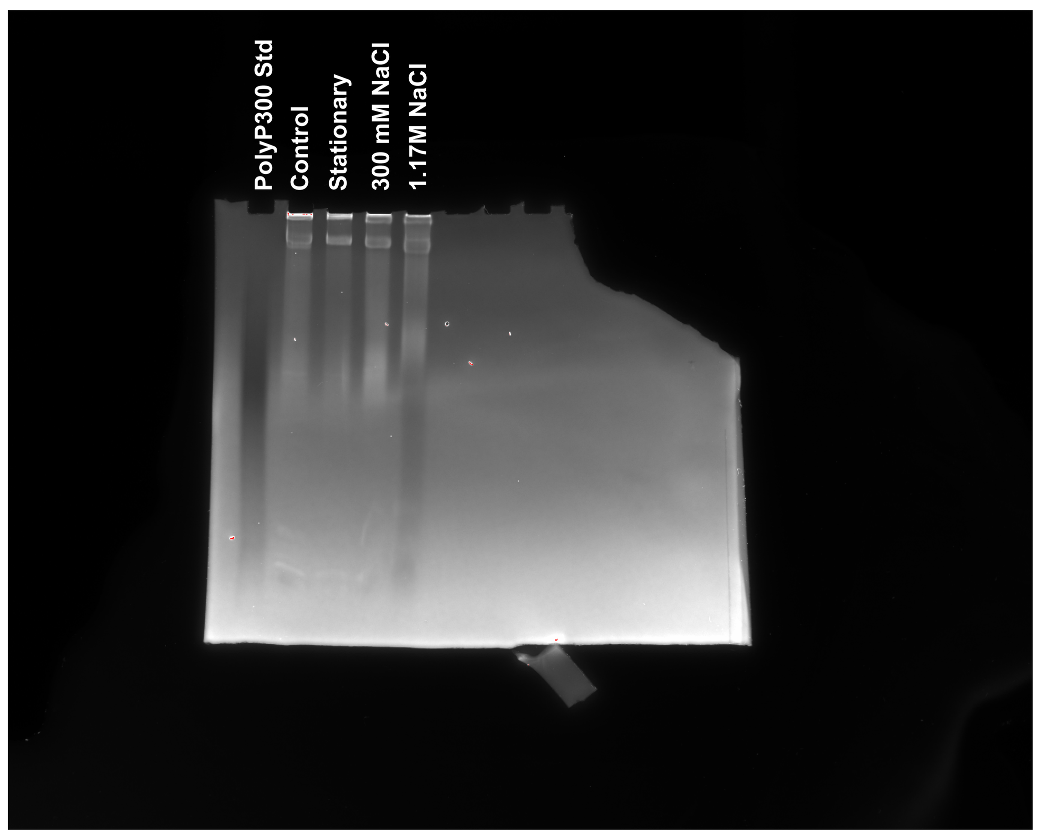

1J

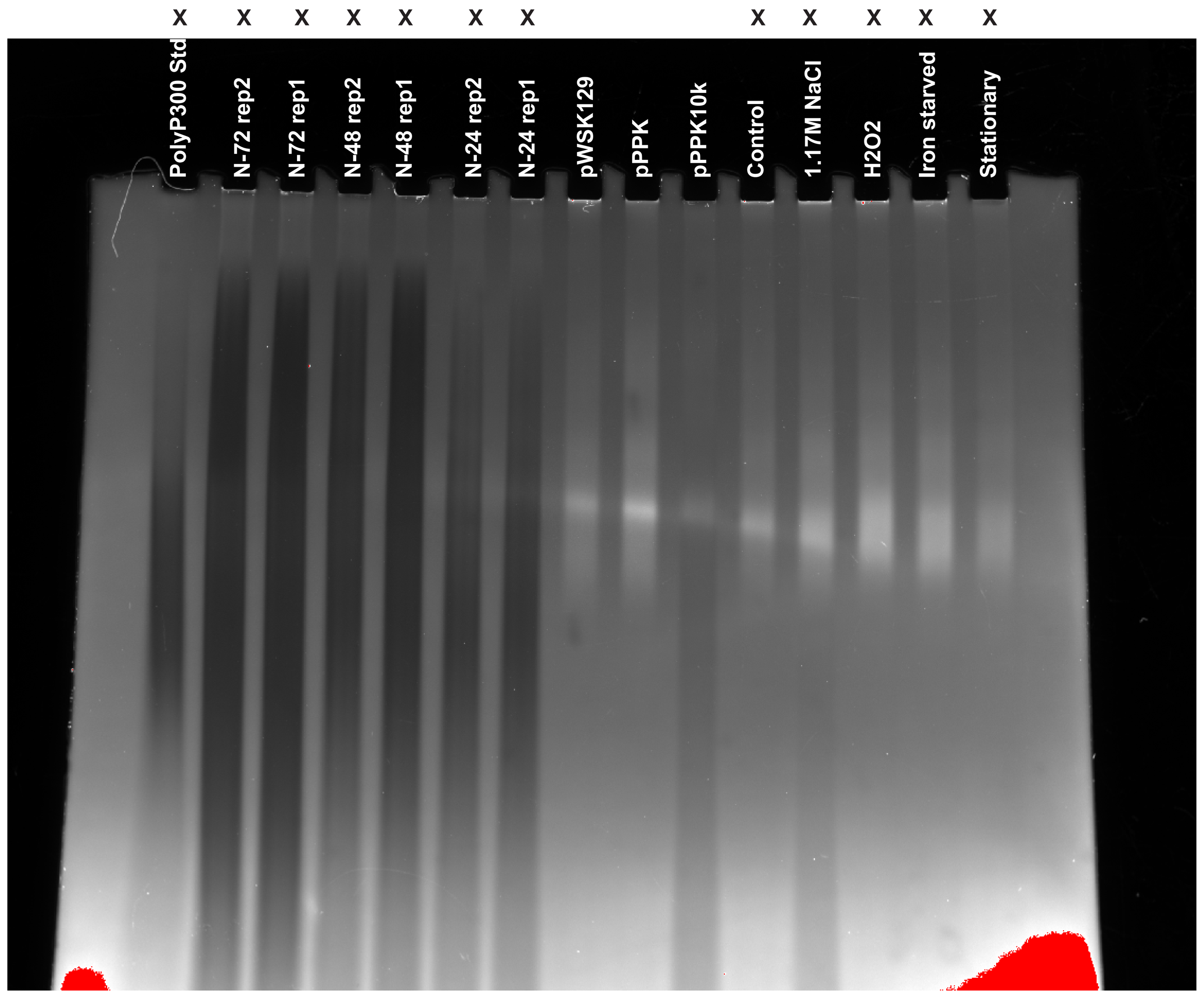

2D

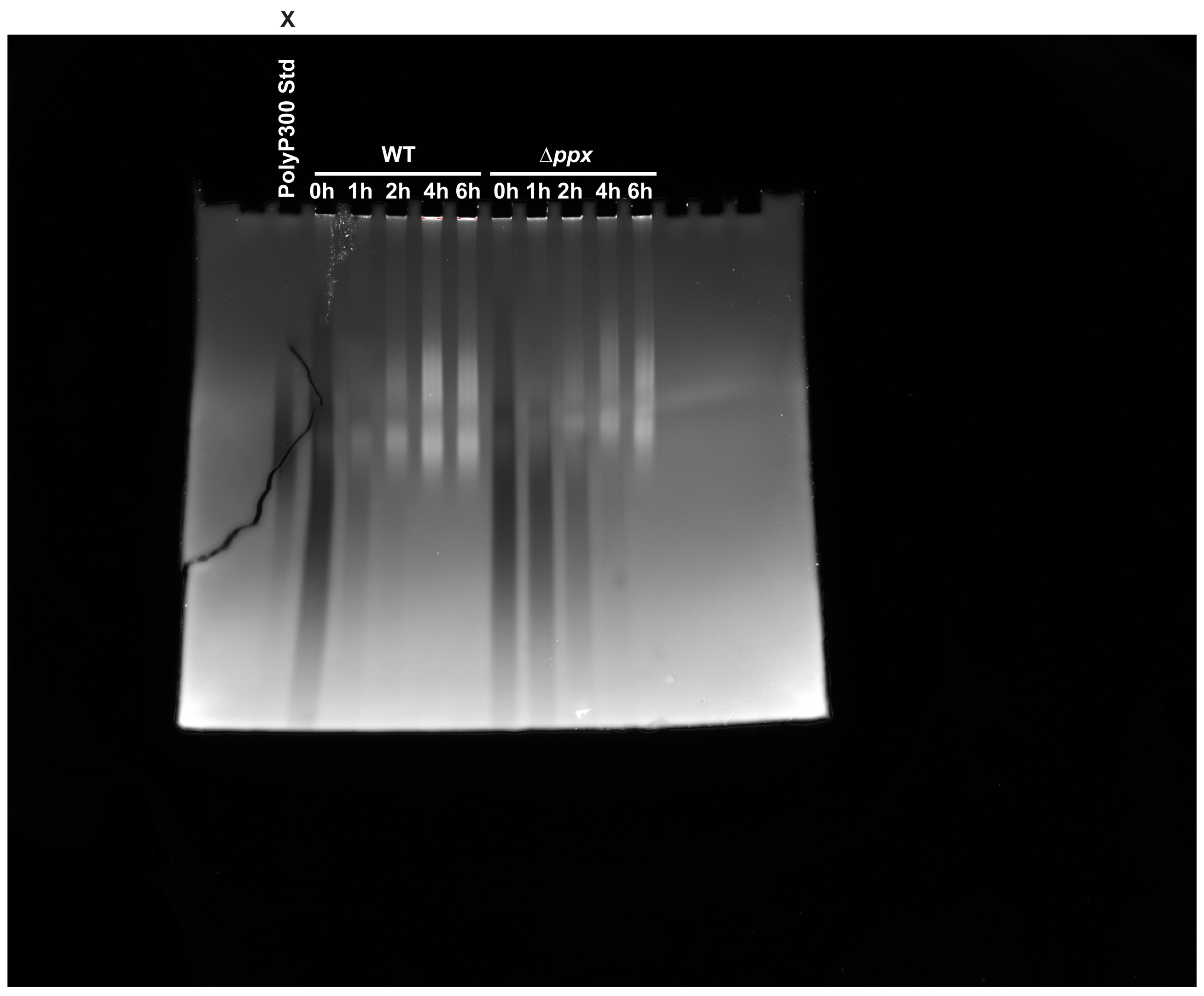

4D PolyP-AF647

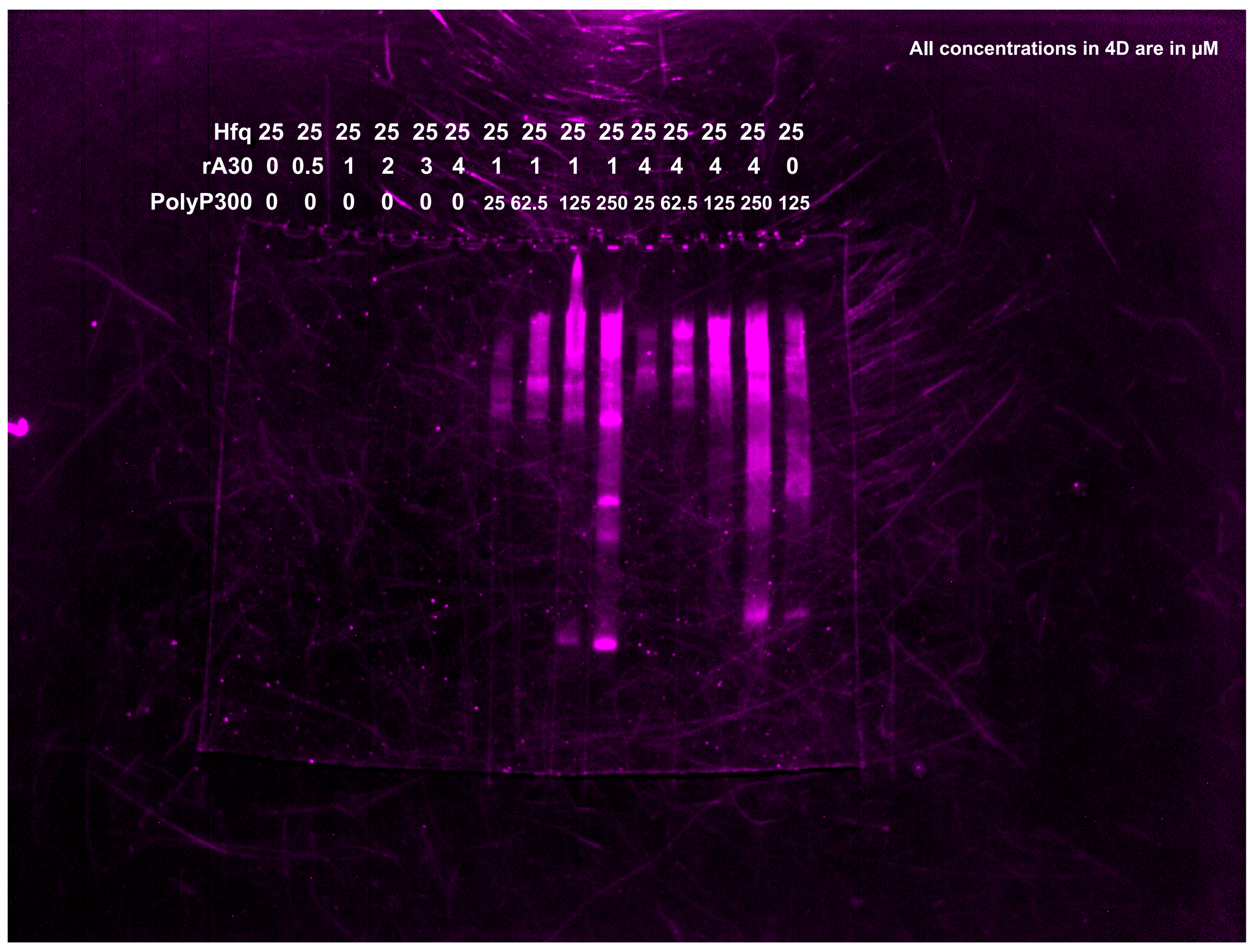

4D rA30-FAM

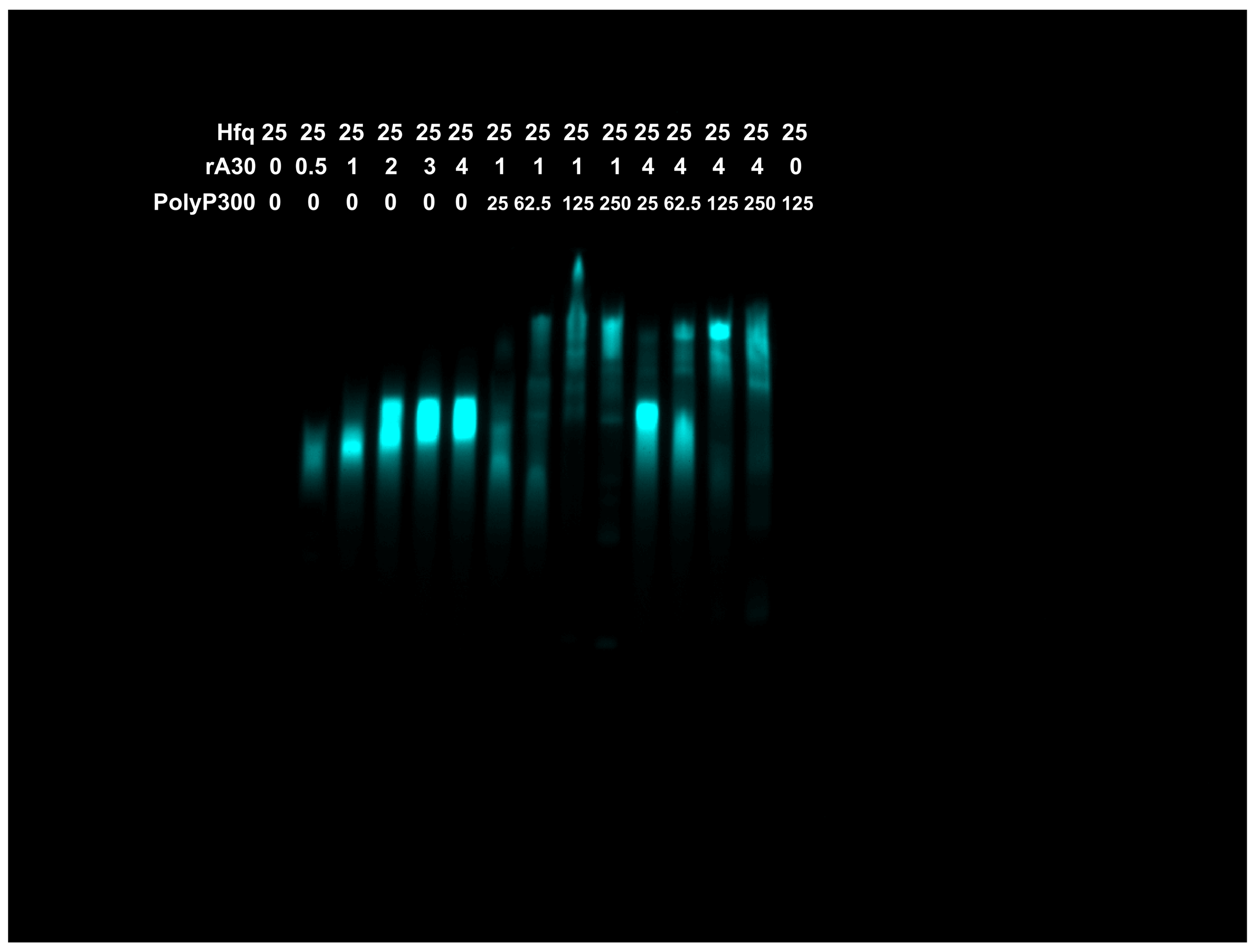

4D Coomassie

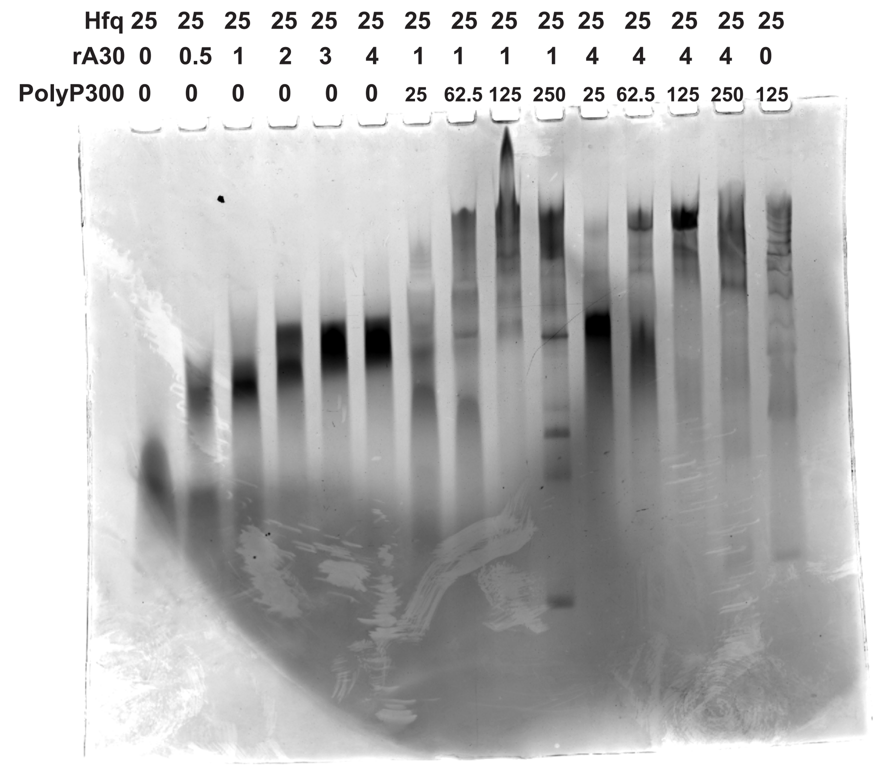

4E/F anti-mCherry

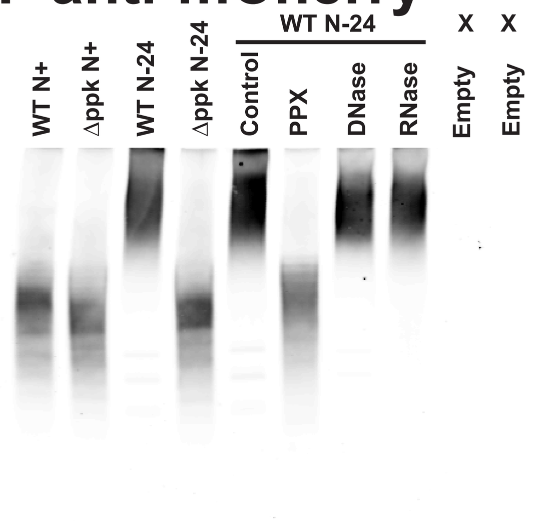

4E/F DAPI

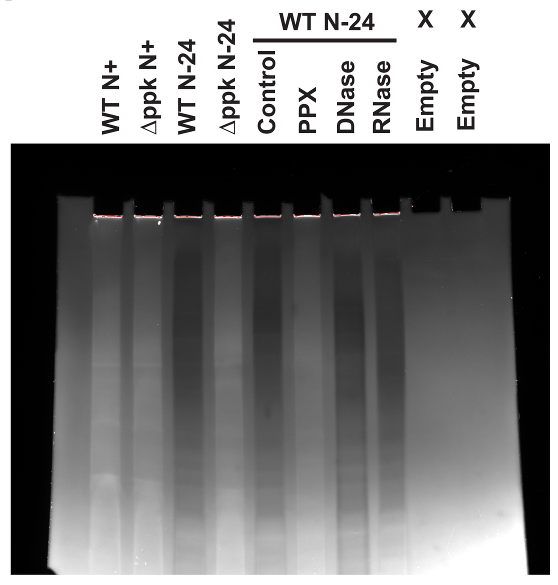

Gel image in 5A is derived from the same one as in 4E/F. Only for the purpose of method illustration.

4G anti-mCherry

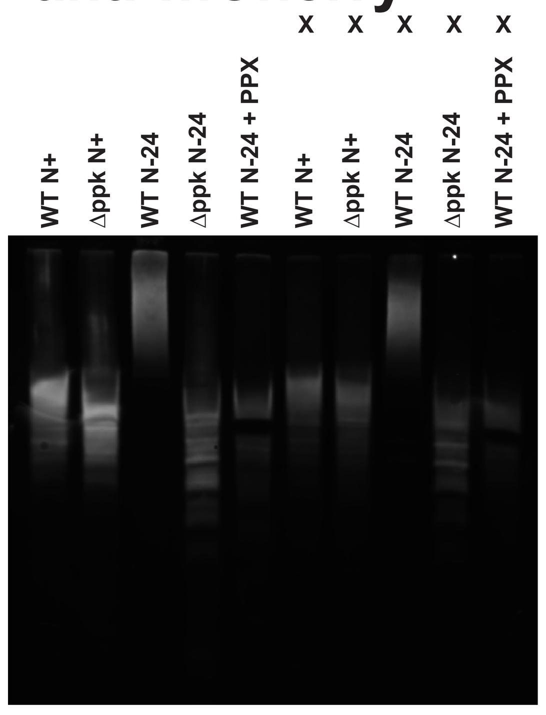

4G anti-GFP (mTurquoise2)

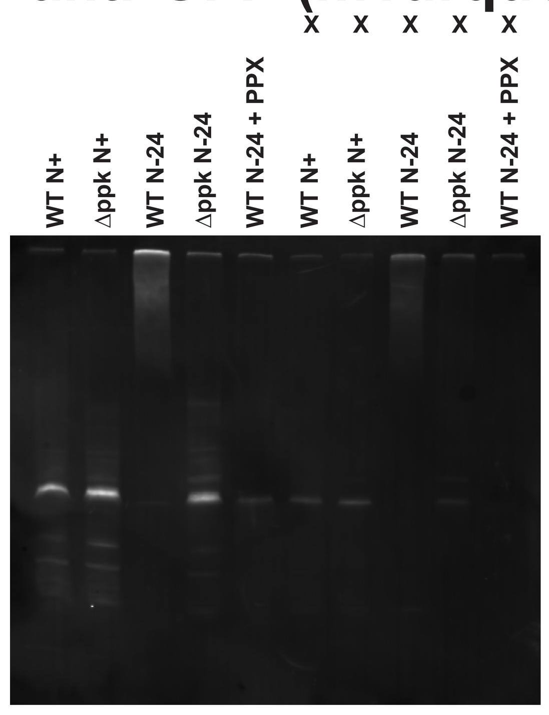

Supplement: S1 Raw Images — (PDF) [file pbio.3003775.s009.pdf]
